# Effectiveness of implementation strategies for digital health implementation in chronic disease care: a systematic review and network meta-analysis

**DOI:** 10.1186/s13012-026-01533-z

**Published:** 2026-08-13

**Authors:** Dorothy Yingxuan Wang, Travis Taihang Shao, Per Nilsen, Olivia Cheri Lam, Zuyao Yang, Kailu Wang, Eliza Lai-Yi Wong, Eng-Kiong Yeoh

**Affiliations:** 1 School of Public Health, Tianjin Medical University, Tianjin, China; 2 JC School of Public Health and Primary Care, Faculty of Medicine, The Chinese University of Hong Kong, Hong Kong SAR, China; 3 Centre for Health Systems & Policy Research, JC School of Public Health and Primary Care, Faculty of Medicine, The Chinese University of Hong Kong, Hong Kong SAR, China; 4 Department of Medical and Health Sciences, Linköping University, Linköping, SE-581 83 Sweden

**Keywords:** Digital health technologies, Implementation strategies, Healthcare professional behavior, Clinical practice

## Abstract

**Background:**

Digital health technologies (DHTs) have proven to be valuable tools in managing chronic conditions. However, significant barriers remain in the implementation of DHTs. A gap remains in understanding the comparative effectiveness of implementation strategies in the context of digital realm. This study aims to make a comprehensive evaluation of the implementation strategies while also investigating potential moderators influencing strategy effectiveness.

**Methods:**

This study is a systematic review and meta-analysis. This study searched four databases and hand searched reference lists from inception to July 2024 for randomized and non-randomized trials with a control group and interrupted time series assessing the impact of at least one implementation strategy to improve DHTs implementation using an outcome that could be mapped to the implementation, patient, and service outcomes based on the Implementation Outcome Framework. Random-effects meta-analysis was conducted. We also conducted meta-regression and subgroup analysis to explore the moderators of the strategy effects and heterogeneity. This study was registered with PROSPERO (CRD42025519493).

**Results:**

We screened 9544 records and selected 32 articles for systematic review and 24 articles were included in the meta-analysis. The most frequently used strategies are “engage consumers”, “support clinicians”, and “train and educate stakeholders”. Most of the articles were with high or serious risk of bias. Implementation strategy as a whole showed a small positive average effect on implementation outcomes compared with no implementation strategy (RD = 0.02, 95% CI: 0.00 to 0.03), but heterogeneity was high (I² = 81.3%). Importantly, after excluding high-risk studies, the pooled effect attenuated to null (RD = -0.00, 95% CI: -0.01 to 0.01) and heterogeneity decreased substantially (I² = 6.6%). The network contained no closed loops, and network meta-analysis estimates were small; 22 of 28 comparative estimates were rated as very low certainty. While the network meta-analysis showed that “train and educate stakeholders” had a relatively favorable effect on implementation outcomes versus no active strategy (RD = 0.03, 95% CI: 0.00 to 0.06, *P* = 0.047), this result was based on low-certainty evidence and should be interpreted cautiously. No strategy was supported by sufficiently certain evidence as superior. The meta-regression indicates that disease type (e.g., cardiac system diseases, diabetes) is an important moderator. The subgroup analysis shows that certain healthcare providers’ behaviors (e.g., documentation, diagnosis) and the number of strategy components play crucial roles in the effects of implementation strategies.

**Conclusions:**

Implementation strategies were associated with a small average improvement in DHT implementation, but effects varied substantially across studies and showed little effects to service or patient outcomes. Given the high risk of bias, substantial heterogeneity, and predominantly low-certainty evidence, the results highlight the importance of tailoring strategies to a specific healthcare context. Future research should also explore long-term effects, multi-level strategies, and sites located in low- and middle-income countries for optimizing DHT implementation.

**Supplementary Information:**

The online version contains supplementary material available at https://doi.org/10.1186/s13012-026-01533-z.

**Contributions to the literature**

- This study examined the effectiveness of implementation strategies to drive healthcare providers to implement the digital health technologies (DHTs) for chronic condition management.
- This study advanced understanding by showing that strategy effectiveness depends on factors such as disease type, targeted healthcare provider behaviors, and number of strategy components, highlighting the need to tailor implementation approaches to specific contexts.
- This study highlighted that given the high risk of bias and heterogeneity across strategy effectiveness, along with limited impact on service and patient outcomes, more reliable data are needed to address the complexity of translating implementation success into health improvements.

## Background

Digital health technologies (DHTs) have enhanced the efficiency and accessibility of care, enabling healthcare systems to better meet the diverse needs of populations [1]. For patients with chronic conditions, DHTs have proven particularly valuable in managing symptoms [2], improving medication adherence [3], and reducing hospital admissions [4]. Healthcare providers also have increasingly embraced DHTs, recognizing their benefits in contributing positively to healthcare professionals’ performance, clinical practice improvements, and greater access to care [5]. 

Encouragingly, global organizations such as the World Health Organization (WHO) have endorsed the broad adoption and scale-up of DHTs [6]. However, the digital health landscape remains complex and fragmented [7]. Key challenges reported by healthcare providers include inadequate infrastructure and technical limitations, psychological resistance, and concerns over increased workloads and longer working hours [8]. Implementation strategies, defined as methods or techniques to promote the initial adoption and sustained use of evidence-based interventions, practices and programs [9], are essential for addressing those challenges. To improve conceptual clarity and comparability across studies, this review was informed by the Expert Recommendations for Implementing Change (ERIC) [10]. The ERIC project developed a refined compilation of 73 discrete implementation strategies and subsequently organized them into nine categories, providing a shared typology for specifying, reporting, and comparing implementation approaches [11]. We used ERIC as the organizing framework for classifying strategies because it is widely used in implementation research and supports more transparent synthesis of heterogeneous interventions.

Despite growing interest in DHTs, there remains a notable gap in research regarding implementation strategies to address implementation barriers and effectively integrate proven DHTs into health systems [12]. Existing systematic reviews and meta-analyses often focus narrowly on individual strategies (e.g., audit and feedback) [13], specific professional disciplines (e.g., nursing) [14] or settings (e.g., low- and middle-income countries) [15], and specific disease groups (e.g., mental health) [16]. Moreover, few have targeted DHTs specifically or addressed the diversity of technologies used across chronic care contexts [17], highlighting the need for more generalizable evidence. Notably, no previous review has employed network meta-analysis (NMA) to rank the relative effectiveness of implementation strategies, making it difficult to determine which strategies should be prioritized in the implementation of DHTs. NMA can compare the average relative effects of strategies across studies, making it a complementary consideration in addition to the clinical and organizational context in devising appropriate implementation strategies. Another important knowledge gap is the limited evidence regarding moderators of strategy effectiveness. Understanding these moderating factors is crucial for informing implementation planning, as it would allow practitioners to deploy strategies at the optimal time and in the most effective manner.

Therefore, this systematic review and meta-analysis aims to evaluate the effectiveness of implementation strategies in supporting healthcare providers implementing DHTs for chronic condition management, as well as on service and patient outcomes, by comparing the relative effects of different types of strategies and examining potential moderators of their effectiveness. Specially, the review seeks to address the following research questions:

1. What is the effect of the implementation strategies on healthcare providers’ implementation of the DHTs and how do different types of implementation strategies compare on average in terms of their effectiveness in improving providers’ implementation of DHTs (primary outcome)?
2. What is the effect of the implementation strategies on service and patient outcomes (secondary outcomes)?
3. What are the potential moderators of the effectiveness of implementation strategies?

## Methods

### Search strategy and selection criteria

The protocol and reporting of this systematic review and meta-analysis adhered to the 2020 PRISMA guidelines [18] and presented in the Supplementary Materials (pp 1–3). We conducted searches on July 4th ,2024 and an updated search on March 16th, 2026, across APA PsycINFO, Ovid Embase, Ovid, MEDLINE, and Scopus, using a wide range of search terms and MeSH terms adapted by previous studies [8, 15, 17]. The search strategy was designed to capture studies related to implementation strategies, DHTs, healthcare providers, chronic diseases, and study design. The full search strategy is provided in the Supplementary Materials (pp 4–17). In addition to the database search, we manually reviewed the reference lists from relevant reviews of digital health technologies in chronic disease management.

We imported citations and abstracts from all retrieved studies into the Endnote 20 software (Clarivate, Philadelphia, PA, USA). Duplicates were removed and two researchers (DYW and OCL) assessed the remaining studies for inclusion, resolving any discrepancies through discussion. The full list of inclusion and exclusion criteria is in the Supplementary Materials (pp 18–19). In brief, we included published peer-reviewed articles with a design of randomized controlled trials (RCTs), cluster RCTs, non-randomized controlled before and after trials, and interrupted time series studies with at least three data points before and after the interventions quantitatively examining the implementation strategies to support healthcare providers’ implementation of DHTs for chronic conditions (e.g., diabetes, heart disease, and depression).

The implementation strategies needed to include at least one intervention component from the nine categories and 73 discrete implementation strategies organized in nine categories in ERIC Taxonomy [11]. Each strategy was mapped according to the definitions of ERIC strategy categories (Supplementary Materials, pp 20–27). It was possible for strategies to be mapped onto more than one sub-category within a single ERIC category. For instance, a single strategy could be categorized under both “Conduct ongoing training” and “Provide ongoing consultation,” which are sub-categories within the broader domain of “Train and educate stakeholders.” Additionally, if a study utilized strategies corresponding to multiple ERIC categories, it was classified as a “multifaceted implementation strategy” in the primary analysis, and its observed effect was not attributed separately to its constituent strategies. Healthcare providers were broadly defined as hospital or outpatient-based health workers; Only articles published in English from inception to March, 2026, were included. For this review, DHTs were defined as digital tools, platforms, or information systems used by, with, or through healthcare providers to support the delivery, documentation, monitoring, communication, or clinical decision-making of chronic disease care. We excluded technologies that were not health-related, were used solely by patients without a provider-facing implementation component, or could not reasonably be classified as part of healthcare delivery or professional practice. Studies had to report at least one objective measure of implementation, service, and patient outcomes (Supplementary Materials, pp 18–19) according to Proctor’s Implementation Outcome Framework [19]. Studies with missing data in total population in group or with time series design were excluded from meta-analysis. The systematic review protocol was registered in PROSPERO CRD42025519493.

### Data extraction

A comprehensive data extraction form was developed based on the Cochrane Handbook for Systematic Reviews of Interventions. This form was piloted on several articles to ensure reliability and reproducibility. Three reviewers (DYW, OLC, and TS) independently extracted the following information from all included studies: publication details (title, year, DOI); authors’ names, study details (country, study objective, study design, study setting, data collection method, sample size), population characteristics (healthcare providers’ role, chronic disease type, healthcare providers and patients’ age, sex, race, and ethnicity), DHTs’ categories, implementation strategy’s characteristics (type, duration, dosage, fully implemented or not, new healthcare providers’ role or responsibility required or not, co-creation or not), comparisons, outcomes (primary and secondary outcomes, outcome categories as implementation, service, and patient outcomes, targeted healthcare providers’ behaviors). Data were extracted and entered into Microsoft Excel (Microsoft Inc, Professional Plus 2016). Discrepancies were resolved through discussion.

### Estimation of effective size

The primary outcome measure, effect size, was defined as an absolute percentage-point difference. We adapted our effect-size calculation approach from Rowe et al. [15]. A positive effect size indicated improvement. For study outcomes in which a decrease indicated improvement, we multiplied the effect sizes by -1. In this study, we included only percentage-based outcomes. For pre-post studies with control groups, the effect size was calculated the difference in improvement over time between the two groups:

$$\begin{aligned}Effect\:size&={\left(Follow\:up-Baseline\right)}_{i}\\ &\quad -{\left(Follow\:up-Baseline\right)}_{c}\end{aligned}$$

*Follow up* can be the proportion with outcome A at follow-up; *Baseline* can be the proportion with outcome A at baseline; i and c denote the implementation-strategy and comparison groups. Meanwhile, many studies did not report baseline information. For these studies, the effect size was calculated as the relative difference in follow-up outcomes between the two groups:

$$\:Effect\:size=\left(\frac{{Follow\:up}_{i}-{Follow\:up}_{c}}{{Follow\:up}_{c}}\right)*100$$

More detailed information for the calculation can be found in the Supplementary Materials (pp 28–29). When a study comparison reported multiple eligible outcomes, the median effect size (MES) of the corresponding effect sizes was used as the estimate of strategy effectiveness for that comparison. Notably, continuous outcomes were assessed but not pooled because their heterogeneous constructs and reporting formats did not support an interpretable common standardized mean difference; these findings were summarized narratively in the Supplementary Materials (pp 56–58).

### Primary analysis

In the primary analysis, a random-effects meta-analysis (which was an a-priori methodological choice) was conducted for the percentage outcomes of the Implementation outcome type to compare the effectiveness of various implementation strategies against no active implementation strategy. Since each study comparison was summarized using a MES, we reported the MES as the risk difference (RD) with a 95% confidence interval (CI). No active implementation strategy was defined as the reference treatment. For multi-arm trials, only the intervention or control groups that met the inclusion or exclusion criteria were included in the analysis. This standard meta-analysis was performed within a frequentist framework using the R package “meta”.

Additionally, to compare the relative effectiveness of different implementation strategies, we conducted a random-effects network meta-analysis (NMA) for the Implementation outcome type. All these analyses were conducted within a frequentist framework using the R package “netmeta”. P-score-based rankings and league table were reported as exploratory summaries of model-estimated relative effects rather than as evidence of strategy superiority. Heterogeneity assessment was also conducted. To consider the plausibility of the transitivity assumption, we descriptively compared the distributions of potential effect modifiers related to study populations, interventions, outcomes, and methodological characteristics across different implementation strategy groups. Consistency assessments (including node-splitting and global inconsistency tests) were not conducted, as the network contained no closed loops; accordingly, comparisons between active strategies and their P-scores were interpreted cautiously alongside heterogeneity, risk of bias, imprecision, and GRADE results.

### Secondary analysis

In the secondary analysis, we conducted a random-effects meta-analysis for the percentage outcomes of the Patient and Service outcome types to obtain the pooled RD and 95% CI. All the secondary analyses were conducted within a frequentist framework using the R package “meta”.

### Assessment of heterogeneity

We assessed heterogeneity across studies using I². We considered heterogeneity to be low if I² < 25%, moderate if I² is between 25% and 75%, and high if I² > 75% [14]. To explore potential sources of heterogeneity among study results, we conducted subgroup analyses and meta-regression. However, due to the limited number of studies, meta-regression was performed only for the primary analysis (i.e., the comparison of implementation strategies vs. no active implementation strategy) to investigate unexplained heterogeneity in RDs between studies. Selected study-level variables were initially examined in separate univariable meta-regression models to explore potential sources of heterogeneity. We then performed bidirectional stepwise selection based on the Akaike information criterion corrected for small sample sizes (AICc), with lower AICc values indicating a better balance between model fit and complexity, to identify a parsimonious meta-regression model. All models were fitted using the same 23 study comparisons and were limited to two moderator terms [20]. For each selected variable, we reported its regression coefficient (B), standard error, 95% CI, and statistical significance in the final model [21]. Given the limited number of comparisons and data-driven selection, findings were interpreted as exploratory. For subgroup analyses, potential effect modifiers were chosen based on the literature review, number of studies available, and the findings from the meta-regression. All analyses were conducted using the R package “meta” and “metafor”.

### Assessment of risk of bias

The revised Cochrane risk-of-bias version 2 (RoB 2) tool was used to assess the quality of the studies of RCTs. For CRTs, risk of bias was assessed with the Cochrane RoB 2 tool for CRTs. For non-randomized controlled trials (NRCS), the risk of bias was assessed with the Risk Of Bias in Non-randomized Studies-of-Interventions (ROBINS-I) tool. RCTs were ranked as having low risk, some concerns, or high risk of bias across five domains, and the overall risk of bias was derived. CRTs were ranked as having low risk, some concerns, or high risk of bias across six domains, and the overall risk of bias was derived. NRCS were ranked as having a low, moderate, serious, or critical risk of bias across seven domains, and the overall risk of bias was derived. Two reviewers (DYW, TS) assessed risk of bias independently, with conflicts resolved through discussion. To identify publication bias, we examined funnel plots and did Egger’s test for our primary analysis [22]. Two authors (DYW, TS) independently assessed the quality of evidence for each outcome using the Grading of Recommendations Assessment, Development, and Evaluation (GRADE) framework [23]. The assessment categorized evidence into four levels (high, moderate, low, or very low) based on five key domains: risk of bias, inconsistency, indirectness, imprecision, and publication bias. In this study, we performed GRADE assessments for all primary and secondary outcomes. Subgroup analyses were exploratory, and separate GRADE assessments were conducted only when a significant interaction between subgroups (subgroup differences P value < 0.05) was identified.

### Sensitivity analysis

We conducted three sensitivity analyses for the primary comparison of implementation strategies versus no active implementation strategy. Some of the included studies were categorized as multifaceted implementation strategy in the primary analysis for the Implementation outcome type, as they incorporated strategies corresponding to multiple ERIC categories. Therefore, in the first sensitivity analysis, we reclassified multifaceted implementation strategies under each ERIC category that they contained. For each ERIC strategy category, this analysis pooled effect estimates from studies in which the evaluated intervention included that category, rather than estimating the independent effect of that category or of an individual strategy. Second, we excluded studies with a high risk of bias to assess their potential impact on the results. We also conducted a more restrictive analysis limited to studies assessed as having low risk of bias. Third, we performed a leave-one-out analysis, sequentially removing one study at a time to determine whether the direction of associations was influenced by a single large study with positive outcomes.

## Results

### Search results

**Fig. 1:**
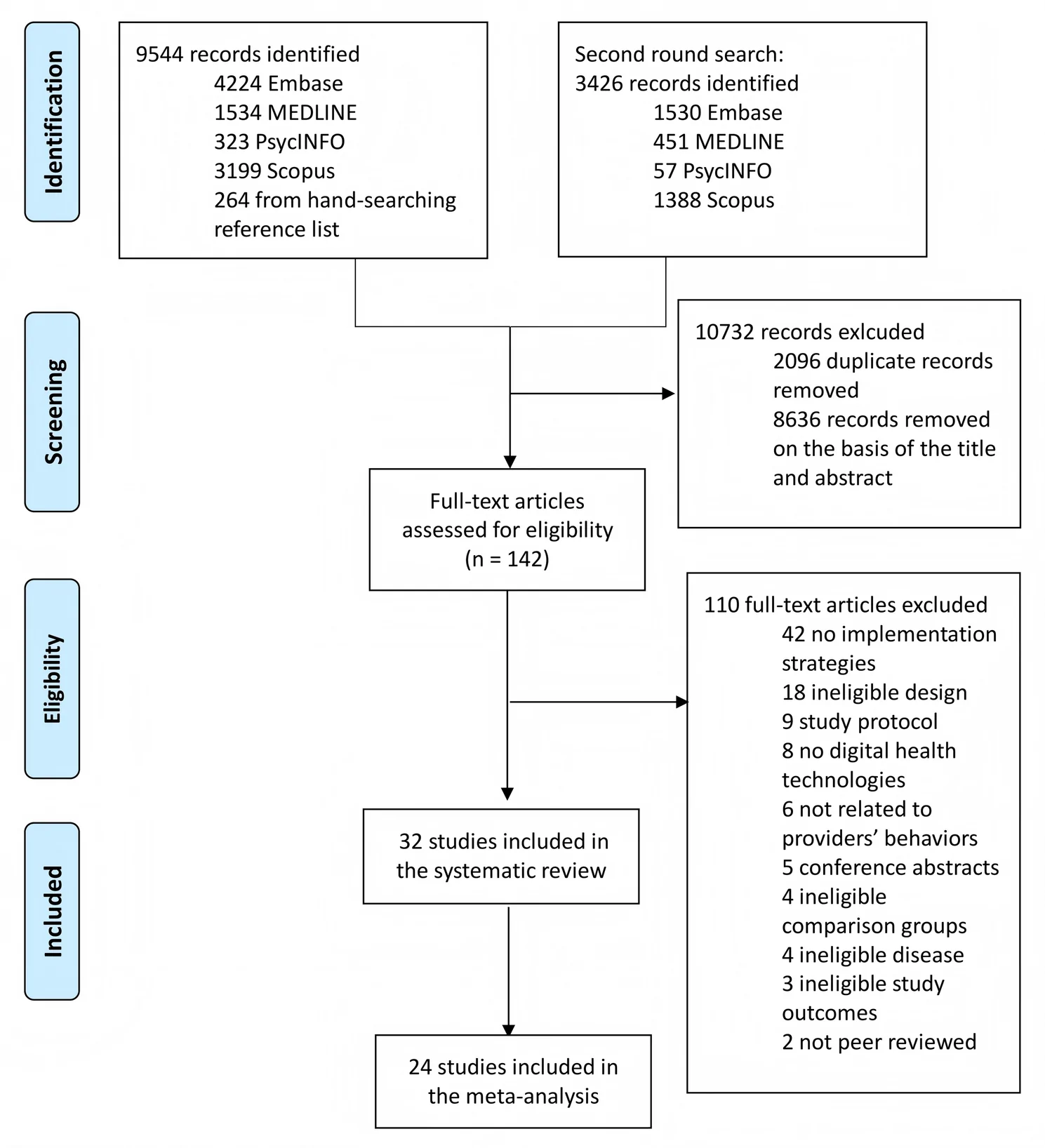
PRISMA flow chart

We searched 9544 articles in the first round and 2436 articles in the second round from electronic databases and hand searching of reference lists (Fig. 1). After excluding 10,732 records, 142 articles were included for full-text screening. Of these studies, 110 were excluded, primarily for an ineligible design and lack of implementation strategies, leaving 32 articles included for the systematic review and 24 articles for meta-analysis. The Supplementary Materials (pp 30–40) includes a list of all full-text excluded studies (*n* = 110) with the justification for exclusion. The Supplementary Materials (pp 56–58) also listed the synthesized results of studies excluded by the meta-analysis.

### Characteristics of included studies

Of 32 included articles (Table 1), all studies were conducted in high-income countries, with 21 originating from the United States. The majority of studies employed a clustered randomized controlled trial design (*n* = 18), followed by traditional randomized controlled trials (*n* = 10). Among the 12 articles that specified geographic location, four studies were conducted in rural areas, seven were from urban areas, and one was from a mixed of rural and urban area. Most of the studies were conducted in outpatient settings (*n* = 22). Thirteen studies took place in public healthcare facilities, while 10 were conducted in private institutions.

**Table 1:** Characteristics of the included studies

Author, Year, Country	Study design	Urban/Rural	Places	Public/Private	HCP Roles	Sample size: intervention	Sample size: control	Chronic conditions	DHT type
Gordon et al. 2022 US	RCT	Rural	Outpatient health facility	Public or government	Physician	Patient: 50	Patient: 51	Diebetes	Telemedicine
Bailey et al. 2020 US	CRT	Not reported	Hospital inpatient wards	Public or government	Muliple HCPs	Facility:24 Providers: 44 Patient: 3414	Facility:23 Providers: 88 Patient: 3078	Multiple chronic conditions	CDSS
Filardo et al. 2009 US	CRT	Rural	Hospital inpatient wards	Private, not for profit	Muliple HCPs	Facility:23	Facility:24	Heart disease	e-Health
Gil-Guillén et al. 2015 Spain	Time series design	Not reported	Outpatient health facility	Public or government	Muliple HCPs	Providers: 941	Providers: 2934	Heart disease	EMR
Sarna et al. 2012 US	RCT	Not reported	Hospital inpatient wards	Unclear or not reported	Nurse	Facility:15	Facility:15	Heart disease	Telemedicine
Estrada et al. 2011 US	CRT	Rural	Outpatient health facility	Unclear or not reported	Physician	Providers: 65 Patient: 715	Providers: 69 Patient: 467	Diebetes	e-Health
Leonard et al. 2024 US	CRT	Not reported	Outpatient health facility	Public or government	Muliple HCPs	Facility:30	Facility:30	Disability	e-Health
Diaz et al. 2021 US	NRCS	Rural	Outpatient health facility	Public or government	Physician	Facility: 1 Provider: 25		Diebetes	Telemedicine
Peels et al. 2024 Netherland	Time series design	Not reported	Outpatient health facility	Public or government	Clinical officer	Facility:8	Facility:16	Physically active decreases	e-Health
Tamblyn et al. 2008 Canada	CRT	Not reported	Outpatient health facility	Public or government	Physician	Providers: 14 Patient: 1899	Providers: 14 Patient: 1550	Multiple chronic conditions	CDSS
van Wyk et al. 2008 Netherland	CRT	Not reported	Outpatient health facility	Public or government	Physician	Facility:13 Providers: 26	Facility:11 Providers: 20	Patients at risk of dyslipidemia	CDSS
Rosenbloom et al. 2005 US	CRT	Urban	Hospital inpatient wards	Private, profit status unknown or not reported	Physician	Providers: 82	Providers: 42	Multiple chronic conditions	Clinical reminder
Strom et al. 2010 US	CRT	Urban	Hospital inpatient wards	Private, profit status unknown or not reported	Muliple HCPs	Providers: 960 Patient: 259	Providers: 1003 Patient: 269	Patients with concurrent orders for warfarin and NSAIDs	EMR
McAlister et al. 2009 Canada	CRT	Not reported	Outpatient health facility	Unclear or not reported	Physician	Patient: 165	Patient: 157	Heart disease	e-Health
Derose et al. 2005 US	RCT	Not reported	Outpatient health facility	Private, not for profit	Physician	Patient: 2311	Patient: 2367	Diebetes	Clinical reminder
Ziemer et al. 2006 US	CRT	Not reported	Outpatient health facility	Public or government	Physician	Patient: 1063	Patient: 1043	Diebetes	Clinical reminder
Phillips et al. 2005 US	CRT	Not reported	Outpatient health facility	Public or government	Physician	Patient: 1063	Patient: 1043	Diebetes	Clinical reminder
Carroll et al. 2018 US	CRT	Not reported	Outpatient health facility	mixed	Physician	Facility: 20 Patient: 5014	Facility: 10 Patient: 1685	CKD	CDSS
Manns et al. 2012 Canada	CRT	Urban+Rural	Outpatient health facility	Unclear or not reported	Physician	Facility: 48 Patient: 2939	Facility: 45 Patient: 2505	CKD	Clinical reminder
Roumie et al. 2006 US	CRT	Not reported	mixed	Public or government	Muliple HCPs	Patient: 470	Patient: 547	Hypertension	Clinical reminder
Loo et al. 2011 US	CRT	Urban	Hospital outpatient department	Private, not for profit	Physician	Patient: 1394	Patient: 1336	Multiple chronic conditions	Clinical reminder
Feldstein et al. 2006 US	RCT	Not reported	Outpatient health facility	Private, not for profit	Physician	Patient: 110	Patient: 101	Osteoporosis	Clinical reminder
Simon et al. 2006 US	CRT	Not reported	mixed	Private, not for profit	Muliple HCPs	Facility: 7	Facility: 8	Multiple chronic conditions	CDSS
Subramanian et al. 2004 US	RCT	Urban	Outpatient health facility	Public or government	Muliple HCPs	Providers: 47 Patient: 355	Providers: 44 Patient: 365	Heart disease	EMR
Heiman et al. 2004 US	CRT	Not reported	Outpatient health facility	Private, not for profit	Physician	Facility:3 Providers: 11 Patient: 385	Facility:2 Providers: 14 Patient: 360	Multiple chronic conditions	Clinical reminder
Blecker et al. 2019 US	RCT	Urban	Hospital inpatient wards	Private, not for profit	Physician	Patient: 465	Patient: 493	Heart disease	CDSS
Leung et al. 2017 Australia	RCT	Urban	Hospital inpatient wards	Unclear or not reported	Physician	Providers: 19	Providers: 20	Multiple chronic conditions	CDSS
Persell et al. 2008 US	CRT	Not reported	Outpatient health facility	Private, not for profit	Physician	Providers: 10 Patient: 177	Providers: 9 Patient: 157	Diabetes	CDSS
Kenealy et al. 2005 New Zealand	CRT	Not reported	Outpatient health facility	Unclear or not reported	Physician	Facility17 Providers: 30 Patient: 4295	Facility:16 Providers: 24 Patient: 4509	Diabetes	Clinical reminder
Stamenova et al. 2023 Canada	RCT	Urban	Hospital outpatient department	Public or government	Muliple HCPs	Patient: 45	Patient: 45	Diabetes	Telemedicine
Schroter et al. 2011 UK	RCT	Not reported	mixed	Unclear or not reported	Physician	Providers: 499	Providers: 498	Diabetes	e-Health
Learmonth et al. 2025 Australia	Time series design	Not reported	Outpatient health facility	Mixed	Muliple HCPs	Providers: 40	Providers: 40	Multiple sclerosis	Telemedicine

In terms of the targeted healthcare professionals, most studies focused on physicians (*n* = 20), followed by multidisciplinary teams (*n* = 10). Regarding the chronic conditions addressed, type 2 diabetes was the most commonly targeted (*n* = 10), followed by cardiovascular disease (*n* = 8). As for the type of DHTs, clinical reminders were most prevalent (*n* = 10), followed by Clinical Decision Support System (*n* = 8) and telemedicine (*n* = 7).

This review identified eight categories of implementation strategies, as classified by the ERIC taxonomy, and one newly categorized implementation strategy (Table 2). The most commonly used strategies were “engage consumers” (*n* = 7) [24–30], “support clinicians” (*n* = 7) [31–37], and “train and educate stakeholders” (*n* = 8) [38–45]. The least applied strategy was “provide interactive assistance” (*n* = 2) [45, 46], with no study focusing on “adapt & tailor to the context”. Most studies employed single strategy (*n* = 25), while seven studies employed a multifaceted implementation strategy. In terms of outcomes, most of studies examined implementation outcomes (*n* = 25), while only seven studies examining patient outcome and four investigating service outcomes.

**Table 2:** Characteristics of the implementation strategies of included articles

Ref	Implementation Strategy Category								Strategy characteristics							
	Engage consumers	Use evaluative & iterative strategies	Change infrastructure	Develop stakeholder interrelationships	Utilize financial strategies	Support clinicians	Provide interactive assistance	Train & educate stakeholders	# of components		Dose	fully implemented?	new HCP responsibilities?	new HCP role?	whether patient co-create	whether HCP co-create
Gordon et al. 2022 US	Prepare patients/consumers to be active participants								1		Once	NR	N	N	Y	Y
Bailey et al. 2020 US								Use train-the-trainer strategies	4		Once	NR	Y	N	N	N
Filardo et al. 2009 US								Conduct ongoing training Provide ongoing consultation	3		> Once	NR	N	N	N	N
Gil-Guillén et al. 2015 Spain								Conduct ongoing training	2		> Once	NR	N	N	N	N
Sarna et al. 2012 US								Make training dynamic	2		NR	NR	N	N	N	N
Estrada et al. 2011 US		Audit and provide feedback				Remind clinicians			6		> Once	NR	N	N	N	Y
Leonard et al. 2024 US				Identify and prepare champions			Facilitation		3		> Once	NR	Y	Y	N	N
Diaz et al. 2021 US					Alter incentive/ allowance structures				1		NA	Y	N	N	N	Y
Peels et al. 2024 Netherland		Audit and provide feedback Develop a formal implementation blueprint		Capture and share local knowledge Build a coalition	Access new funding		Centralize technical assistance	Develop and Distribute educational materials Conduct educational meetings	9		> Once	NR	Y	N	N	Y
Tamblyn et al. 2008 Canada			Change physical structure and equipment						1		NA	NR	N	N	N	N
van Wyk et al. 2008 Netherland						Remind clinicians			1		NA	Y	N	N	N	N
Rosenbloom et al. 2005 US						Remind clinicians			1		NA	NR	N	N	N	N
Strom et al. 2010 US			Enable choice						1		NA	NR	N	N	N	N
McAlister et al. 2009 Canada				Inform local opinion leaders					1		NA	NR	N	N	N	N
Derose et al. 2005 US			Enable choice						1		NA	NR	N	N	N	N
Ziemer et al. 2006 US		Audit and provide feedback							1		> Once	NR	N	N	N	N
Phillips et al. 2005 US		Audit and provide feedback							1		> Once	NR	N	N	N	N
Carroll et al. 2018 US		Audit and provide feedback		Identify and prepare champions Obtain formal commitments Use an implementation advisor Organize clinician implementation team meetings					5		> Once	NR	Y	Y	N	NR
Manns et al. 2012 Canada						Remind clinicians			1		NA	Y	N	N	N	N
Roumie et al. 2006 US	Involve patients/consumers and family members								4		NR	NR	N	N	N	N
Loo et al. 2011 US						Revise professional roles			1		> Once	NR	Y	Y	N	N
Feldstein et al. 2006 US	Involve patients/consumers and family members								1		NR	NR	N	N	N	N
Simon et al. 2006 US				Identify and prepare champions				Use train-the-trainer strategies Conduct educational meetings Distribute educational materials	4		NR	NR	Y	N	N	Y
Subramanian et al. 2004 US						Remind clinicians			1		NA	NR	N	N	N	Y
Heiman et al. 2004 US	Prepare patients/consumers to be active participants								1		Once	NR	N	N	N	N
Blecker et al. 2019 US						Remind clinicians			1		NA	NR	N	N	N	Y
Leung et al. 2017 Australia		Audit and provide feedback						Distribute educational materials Conduct educational meetings	1		NR	NR	N	N	N	N
Persell et al. 2008 US	Prepare patients/consumers to be active participants								2		NR	NR	Y	N	N	N
Kenealy et al. 2005 New Zealand	Prepare patients/consumers to be active participants								1		NR	NR	N	N	N	N
Stamenova et al. 2023 Canada	Prepare patients/consumers to be active participants Involve patients/consumers and family members								1		Once	NR	Y	N	N	N
Schroter et al. 2011 UK		Conduct local need assessment							1		Once	NR	Y	NR	N	N
Learmonth et al. 2025 Australia							Provide local technical assistance	Distribute educational materials	2		> Once	NR	Y	N	N	Y

Abbreviations: NR = Not reported; NA = Not applicable; HCP=Healthcare Provider; Y = Yes; N = No

### Risk of bias

We summarized the decisions regarding individual domains within the Cochrane RoB 2 tool and ROBINS-I in the Supplementary Materials (pp 43–44). Overall, for CRT, the risk of bias for all outcome assessments was distributed as follows: 5 (28%) were assessed as low risk, 3 (17%) had some concerns, and 10 (56%) were considered high risk. For RCTs, 2 (20%) of the assessments were categorized as having some concern and 8 (80%) as high risk. For NRCS, 4 (100%) were considered to have a serious risk.

### Primary analysis for the implementation outcomes

Table 3 shows the strategies’ effectiveness on implementation outcomes in all the 23 included studies. Table 3 also indicates the certainty of evidence (GRADE) for each type of strategy effectiveness compared to no active implementation strategy. Detailed GRADE scoring process and results are presented in the Supplementary Materials (pp 45–51).

**Table 3:** Effectiveness of implementation strategies to enhance implementation of digital health technologies

Comparison	Number of study	Pooled MES RD [95%CI]	Evidence source	GRADE quality of evidence
**Standard meta-analysis (Primary analysis)**				
Implementation strategy vs. no active implementation strategy (Implementation)	23	0.02 [0.00; 0.03]	Direct evidence	low
***Standard meta-analysis (Secondary analysis***,*** other outcome types)***				
Implementation strategy vs. no active implementation strategy (Patient)	3	-0.01 [-0.03; 0.02]	Direct evidence	moderate
Implementation strategy vs. no active implementation strategy (Service)	2	-0.03 [-0.09; 0.04]	Direct evidence	moderate
***Network meta-analysis (Primary analysis)***				
Change infrastructure vs. Multifaceted implementation strategy	0	-0.02 [-0.08; 0.04]	Indirect evidence	very low
Change infrastructure vs. Develop stakeholder interrelationships	0	-0.06 [-0.21; 0.09]	Indirect evidence	very low
Change infrastructure vs. Engage consumers	0	0.01 [-0.06; 0.08]	Indirect evidence	very low
Change infrastructure vs. No active implementation strategy	2	0.00 [-0.04; 0.04]	Direct evidence	low
Change infrastructure vs. Support clinicians	0	-0.02 [-0.07; 0.02]	Indirect evidence	very low
Change infrastructure vs. Train & educate stakeholders	0	-0.03 [-0.08; 0.20]	Indirect evidence	very low
Change infrastructure vs. Use evaluative & iterative strategies	0	0.01 [-0.05; 0.08]	Indirect evidence	very low
Multifaceted implementation strategy vs. Develop stakeholder interrelationships	0	-0.04 [-0.19; 0.10]	Indirect evidence	very low
Multifaceted implementation strategy vs. Engage consumers	0	-0.03 [-0.05; 0.10]	Indirect evidence	very low
Multifaceted implementation strategy vs. No active implementation strategy	3	0.02 [-0.03; 0.06]	Direct evidence	low
Multifaceted implementation strategy vs. Support clinicians	0	0.01 [-0.06; 0.04]	Indirect evidence	very low
Multifaceted implementation strategy vs. Train & educate stakeholders	0	-0.01 [-0.06; 0.04]	Indirect evidence	very low
Multifaceted implementation strategy vs. Use evaluative & iterative strategies	0	0.03 [-0.04; 0.10]	Indirect evidence	very low
Develop stakeholder interrelationships vs. Engage consumers	0	0.07 [-0.08; 0.23]	Indirect evidence	very low
Develop stakeholder interrelationships vs. No active implementation strategy	1	0.06 [-0.08; 0.20]	Direct evidence	moderate
Develop stakeholder interrelationships vs. Support clinicians	0	0.04 [-0.11; 0.18]	Indirect evidence	very low
Develop stakeholder interrelationships vs. Train & educate stakeholders	0	0.03 [-0.11; 0.18]	Indirect evidence	very low
Develop stakeholder interrelationships vs. Use evaluative & iterative strategies	0	0.07 [-0.08; 0.22]	Indirect evidence	very low
Engage consumers vs. No active implementation strategy	6	-0.01 [-0.07; 0.05]	Direct evidence	moderate
Engage consumers vs. Support clinicians	0	-0.03 [-0.10; 0.03]	Indirect evidence	very low
Engage consumers vs. Train & educate stakeholders	0	-0.04 [-0.11; 0.03]	Indirect evidence	very low
Engage consumers vs. Use evaluative & iterative strategies	0	0.00 [-0.08; 0.08]	Indirect evidence	very low
Support clinicians vs. No active implementation strategy	6	0.02 [-0.00; 0.05]	Direct evidence	very low
Train & educate stakeholders vs. No active implementation strategy	4	0.03 [0.00; 0.06]	Direct evidence	low
Use evaluative & iterative strategies vs. No active implementation strategy	1	0.01 [-0.04; 0.06]	Direct evidence	moderate
Support clinicians vs. Train & educate stakeholders	0	-0.00 [-0.04; 0.03]	Indirect evidence	very low
Support clinicians vs. Use evaluative & iterative strategies	0	0.04 [-0.02; 0.09]	Indirect evidence	very low
Train & educate stakeholders vs. Use evaluative & iterative strategies	0	0.04 [-0.02; 0.10]	Indirect evidence	very low

MES=median effect size; see Methods section for more details on the calculation process; GRADE=Grading of Recommendations Assessment, Development, and Evaluation system

#### Effects of implementation strategies compared to no active implementation strategy

**Fig. 2:**
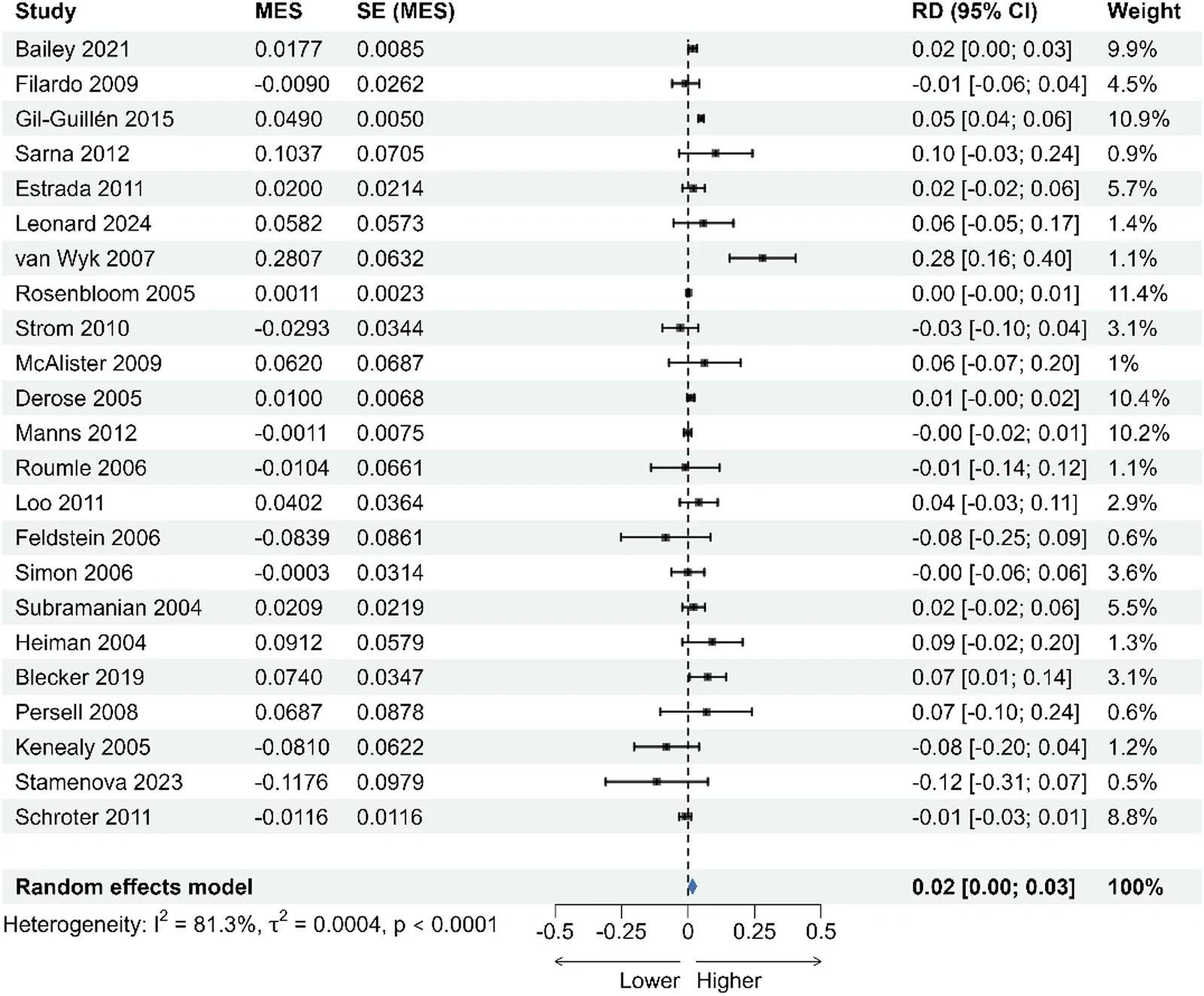
Standard Meta-analysis on the overall effects of implementation strategy versus no active implementation strategy on implementation outcomes. MES=Median effect size; SE=Standard error; RD=Risk difference; CI=Confidence interval

The random-effects model estimated a pooled RD of 0.02 [0.00, 0.03], indicating a small average improvement in implementation outcomes with implementation strategies compared with no active implementation strategy (*p* = 0.02) (Fig. 2). However, effects varied substantially across studies (I² = 81.3%, *p* < 0.0001), suggesting considerable variability across studies.

#### Relative effects of different implementation strategies

**Fig. 3:**
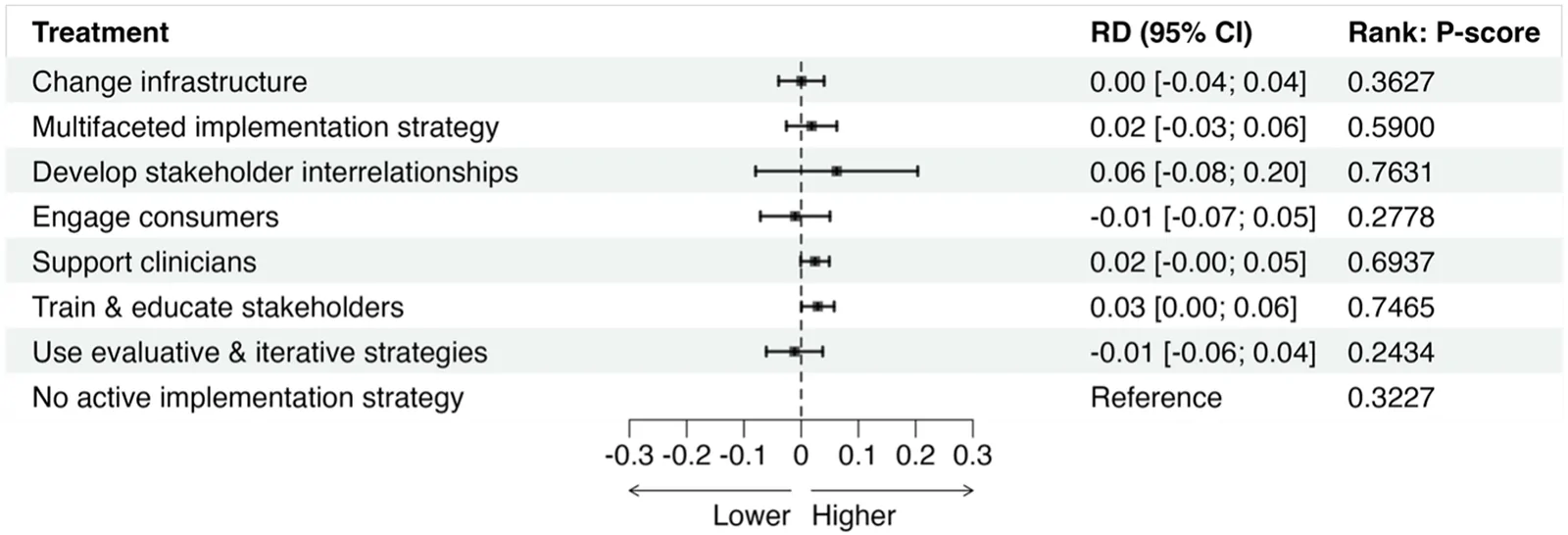
Network meta-analysis on the effect of implementation strategy on implementation outcomes. “Multifaceted implementation strategy” denotes study arms that included strategies from more than one ERIC strategy category. In the primary analysis, each package was entered once as a package-level comparison. The three packages comprised: “use evaluative and iterative strategies” combined with “support clinicians”; one study applying “provide interactive assistance” combined with “develop stakeholder interrelationships”, and one study applying “train & education stakeholders” combined with “develop stakeholder interrelationships”. P-scores summarize the model-based ranking of strategies and do not represent certainty of evidence or proof of superiority. The network contained no closed loops; therefore, comparisons between active strategies rely on the transitivity assumption and should be interpreted alongside the confidence intervals, risk-of-bias assessments, heterogeneity, and GRADE. RD=Risk difference; CI=Confidence interval

The forest plot and network plot illustrated the structure of the comparisons, which can be found as Fig. 3 and in the Supplementary Materials (pp 52). The network contained no closed loops. The results of the random-effects NMA model indicated that among the evaluated strategies, “train and educate stakeholders” (RD = 0.03 [0.00, 0.06], *p* = 0.0470) demonstrated a small positive effect compared to no active implementation strategy. This direct comparison was informed by four studies, all at high risk of bias, and was rated as low-certainty evidence. Point estimates were positive for all the other strategies except “Engage consumers” and “Use evaluative & iterative strategies”, but with substanital levels of uncertainty. The P-score-based ranking analysis (Fig. 3) placed “develop stakeholder interrelationships” the highest. However, this intervention was evaluated in only one study, which limits the credibility of this finding. The league table showed a small positive effect for “train and educate stakeholders” compared with “no active implementation strategy” (Supplementary Materials, pp 53). These rankings are exploratory and should not be interpreted as evidence of superiority.

The heterogeneity assessment (Supplementary Materials, pp 54) revealed high variability across studies, with the Q statistic for total heterogeneity being 49.64 (df = 16, *p* < 0.0001). To be specific, the heterogeneity was highest in comparisons involving “support clinicians” (Q = 25,93, df = 5, *p* < 0.0001) and “train and educate stakeholders” (Q = 14.52, df = 5, *p* = 0.0023).

According to the GRADE assessment, the certainty of evidence for the NMA estimates was rated as moderate for 3 comparisons (10.7%), low for 3 comparisons (10.7%), and very low for 22 comparisons (78.6%). The majority of the NMA comparisons was informed solely by indirect evidence, and most were downgraded due to imprecision and intransitivity.

### Secondary analysis for the patient and service outcomes

Supplementary Materials (pp 55) presents the results of the outcome type of service and patient. Compared with “no active implementation strategy”, implementation strategies did not show a clear difference in either outcomes. The pooled RD was − 0.01 [-0.03, 0.02] for patient outcomes and − 0.03 [-0.09, 0.04] for service outcomes. Additionally, the heterogeneity was low-to-moderate in these two models (I² = 32% and 0.0% respectively).

### Meta-regression and subgroup analysis

A total of 17 variables were considered in the meta-regression (Supplementary Materials, pp 59–61). In the bidirectional stepwise selection based on AICc, disease category was selected as the most parsimonious moderator model. No additional moderator improved model fit after disease category was included. Compared with cardiac system diseases, estimated effects were smaller for diabetes (β = -0.04, [-0.06, -0.03]), mixed disease populations (β = − 0.03, [-0.05, -0.01]), and other conditions (β = − 0.05, [-0.06, -0.04]).

Four effect modifiers were analysed in subgroup analyses: healthcare providers’ behaviours, disease category, number of strategy components, and intervention frequency (Supplementary Materials, pp 62–65). Subgroup effect was observed by studies targeting different healthcare providers’ behaviours (*p* < 0.0001) and number of strategy components (*p* = 0.02). Documentation (e.g., record smoking and alcohol status in the EMR) and diagnosis (e.g., diagnosis of hypertension based on clinical guidelines) showed a greater effect (RD = 0.05 [0.04; 0.06] and 0.05 [0.04; 0.06] respectively) compared with other healthcare providers’ behaviours. Studies involving more than one strategy component showed a larger positive effect (RD = 0.03 [0.01; 0.05]) than one strategy component only. Although the univariable meta-regression suggested a significant association between disease category and effect size, the subgroup analysis did not confirm the between-group differences (*p* = 0.07).

### Publication bias

For the standard meta-analysis in the primary analysis, the funnel plot exhibited symmetry, and the Egger’s test showed that there is no publication bias (*p* = 0.3249) (Supplementary Materials, pp 66). The funnel plot for the NMA also appeared symmetrical, and the Egger’s test did not indicate significant publication bias (*p* = 0.1) (Supplementary Materials, pp 67). This suggested that the observed effect in both the standard meta-analysis and the NMA results were less influenced by selective reporting.

### Sensitivity analysis

After reclassifying multifaceted implementation strategies under their constituent ERIC categories, six additional category-level entries were included in the analysis (one for “train & educate stakeholders”, one for “support clinicians”, two for “develop stakeholder interrelationships”, one for “provide interactive assistance”, and one for “use evaluative & iterative strategies”). These entries represent the implementation strategy that included the respective ERIC category, rather than independent evaluations of individual strategies. The results indicated that compared with the primary analysis, the overall conclusion based on the pooled estimates remained unchanged (Supplementary Materials, pp 68). After removing high-risk studies, the number of included studies decreased from 23 to 7 (Supplementary Materials, pp 69). The RD changed from 0.02 [0.00, 0.03] to -0.00 [-0.01, 0.01]. The heterogeneity decreased from I² = 81.3% to 6.6%. In the more restrictive analysis limited to three low-risk studies, the pooled RD was 0.03 (95% CI: -0.02 to 0.09). Although no heterogeneity was detected (I² = 0.0%), the estimate was imprecise and its confidence interval included the null. The leave-one-out sensitivity analysis revealed that when most studies were omitted, the RD estimates remained consistent, ranging from approximately 0.0068 to 0.0192, and the heterogeneity remained high (I² ≈ 52.9% to 82.2%) (Supplementary Materials, pp 69–70). However, omitting the study conducted by Gil-Guillén et al. led to a substantial reduction in heterogeneity (I² = 52.9%) and resulted in a notably lower pooled RD of 0.01 [-0.0008; 0.01].

## Discussion

This systematic review synthesized the evidence from 32 studies on the effectiveness of the implementation strategies supporting healthcare providers implementing the DHTs for chronic disease management. Overall, the meta-analysis of 24 studies suggest that implementation strategies may lead to modest, context-dependent improvements in implementation outcomes rather than large or uniform gains across settings. However, after excluding high-risk studies, the pooled effect attenuated to null and heterogeneity decreased markedly, suggesting that the apparent positive overall effect may be driven largely by studies with greater methodological limitations. The estimates for patient and service outcomes were close to null. Furthermore, meta-regression revealed that the disease type could moderate the effects of the implementation strategies. The subgroup analysis showed that certain healthcare providers’ behaviours (i.e., documentation and diagnosis) and intense strategy design (more than one strategy component) showed greater effect in the application of implementation strategies. However, the current evidence base still lacks robustness due to the high risk of bias and heterogeneity present across the included studies.

At the level of specific implementation strategies, “train and educate stakeholders” appeared to have one of the more favorable estimated effects, which is consistent with previous reviews emphasizing training as a way to improve knowledge, technical confidence, and perceived feasibility of new technologies [15, 16, 21]. Previous evidence showed that training has been demonstrated to increase clinicians’ technical skills and alter perceptions of complexity and benefit of the technologies [47], therefore leading to higher intentions to use the DHTs based on Theory of Planned Behavior [48]. However, the estimated effect was modest, heterogeneity was high, and the certainty of evidence was low, suggesting that policymakers and clinical leaders should interpret these findings with caution and avoid making definitive decisions based solely on uncertain evidence. Furthermore, the variation across the included studies suggests that “train and educate stakeholders” should be understood in terms of core functions and adaptable forms rather than as a fixed strategy package. Core functions represent the theoretical mechanisms or processes that must remain consistent to preserve effectiveness, whereas adaptable forms can flexibly accommodate local contexts, needs, and resources [49]. For example, the core function of included studies is to develop the knowledge and skills required to perform the targeted clinical behavior while the forms used to enact these functions may be adapted to local needs and resources, including the delivery modality, duration and intensity of training, type of trainer, degree of interactivity, educational materials, and nature and frequency of follow-up support [38–41]. Additionally, using structured adaptation frameworks such as Framework for Reporting Adaptations and Modifications to Evidence-based Implementation Strategies (FRAME-IS) helps systematically document what elements are adapted, why adaptations are needed, and how they align with core functions [50], therefore enhancing transparency of the implementation process and promoting replicability and scalability.

In our analysis, although implementation strategies resulted in modest improvements in implementation outcomes, they did not show measurable effects on service quality or patient outcomes. This disparity can be explained by that the implementation strategies tested may have been insufficiently intensive to change clinical care in ways that would translate into service or patient benefit, and some implementation outcomes measured in the included studies—such as documentation rates or system-use metrics—may be imperfect proxies for the clinical behaviors that actually influence patient outcomes. In addition, implementation outcomes may be necessary but not sufficient conditions for improving clinical or service outcomes [19]. For instance, some studies have found that hospitals with high adherence to process indicators do not show significant differences in clinical outcomes compared to hospitals with lower adherence rates [51]. The reason for this may lie in the complexity of factors influencing patient outcomes, which cannot be solely addressed by improving process measures [14]. Furthermore, the effects of implementation strategies on service and patient outcomes often require time to emerge. Many studies included in this meta-analysis had relatively short follow-up periods, potentially limiting the ability to detect long-term effects. These findings highlight the need for linking implementation outcomes to clinically meaningful behavioral change, more robust, multi-level implementation strategies and longer-term studies to fully understand the effects of DHT implementation on patient health and service outcomes.

In our analysis, meta-regression suggested that disease type may moderate the outcomes, where studies in diabetes or multiple‐disease settings had lower effects than those in cardiovascular care. Subgroup analyses likewise showed that studies targeting certain provider behaviors, such as documentation or diagnostic practices, yielded larger effects, with absolute differences of about 5% points, as did those using multi‐component strategies, with an estimated difference of about 3% points, aligning with prior evidence that bundling strategies and tailoring them to local barriers tend to produce more robust implementation gains [14]. These magnitudes remain modest, but they may still be meaningful when the targeted behavior occurs at scale, the strategy is relatively low cost, and even small gains are expected to accumulate across large patient populations. At the same time, effects of this size may be less meaningful when implementation requires substantial resources or when downstream patient and service benefits are not demonstrated. Future studies should therefore examine the cost-effectiveness of the strategy and whether the effect is sustained over time.

High heterogeneity reflects real-world complexity and limits simple pooling [17], suggesting that one-size-fits-all recommendations may not be tenable. Multi-component, behavior‐targeted strategies can be emphasized in practice, while policy frameworks should allow adaptable, locally tailored implementation support [52]. Multi-component strategies should be developed by identifying determinants operating at different levels, followed by selection of corresponding strategy components targeting those barriers. Powell et al. proposed systematic approaches for linking identified barriers and facilitators to tailored implementation strategies, providing a practical basis for designing such multilevel strategy packages [53]. For behaviour-targeted implementation, the Behaviour Change Wheel provides a practical approach: specify who needs to do what differently and in which context, identify barriers through methods such as interviews, observation, or audit data, and then select intervention functions that address the relevant capability, opportunity, and motivation determinants [54]. Researchers are suggested to design stratified or factorial trials and report detailed context. Factorial trial designs such as the Multiphase Optimization Strategy (MOST) can efficiently test multiple intervention components or strategies in parallel, making it easier to identify which combinations work best for which subgroups [55]. Policy‐makers and funders should support pragmatic yet rigorous studies of DHT implementation that build in heterogeneity and explicitly test how strategy effects vary by context to develop tailored and equitable DHT implementation strategies.

Our study has several limitations that should be considered when interpreting the findings. First, we limited our focus to experimental studies only, leaving out many valuable observational studies that could have provided a more comprehensive exploration of various implementation strategies. Second, most of the included studies did not use the ERIC taxonomy to define their implementation strategies. As a result, we retrospectively mapped the strategies to the taxonomy, which could introduce inaccuracies. However, we addressed discrepancies between the two coders and consulted a third senior implementation researcher to minimize potential misunderstandings. Third, many of the included studies lack details on the strategy and context, employ diverse delivery approaches, and have a high risk of bias, which may compromise the validity of the meta-analysis results. Fourth, our categorization of implementation strategies may have resulted in some important nuances being overlooked in the final results. For instance, while we grouped all clinician support strategies together based on their common goal of supporting clinicians, these strategies may differ in form (e.g., reminders, workflow redesign). Future analyses could benefit from more specific classifications to better capture these variations. Fifth, all included studies were conducted in high-income countries. As a result, the current evidence mainly reflects settings with relatively stronger digital infrastructure, workforce capacity, financing arrangements, and institutional support for implementation. The feasibility and effectiveness of strategies may differ in low- and middle-income countries or other resource-constrained settings, where infrastructure gaps, staffing shortages, and different policy environments may shape both implementation barriers and responses to implementation strategies. Therefore, the generalizability of our findings beyond high-income settings is uncertain. Sixth, although we screened whether strategies were reported as fully implemented, most studies did not report adherence to protocol, adaptations, or fidelity in sufficient detail to support a reliable assessment. This limited our ability to determine whether small or null effects reflected differences in implementation quality. Future DHT implementation studies should therefore report strategy fidelity, dose, uptake, and adaptations more systematically. Seventh, the evidence network for the NMA contained no closed loops. As a result, formal consistency assessment was not possible, and the comparative estimates and rankings were based predominantly on indirect evidence that depended on the plausibility of transitivity. Although some potential effect modifiers were comparable across strategy groups, including country income level and outcome measurement, the other factors, such as disease category, were unevenly distributed. Therefore, the plausibility of the transitivity assumption could not be fully established. Accordingly, the NMA findings were treated as exploratory rather than as definitive evidence of strategy superiority, and the certainty of relevant estimates was downgraded. Subgroup, sensitivity, and meta-regression analyses were also conducted to further explore heterogeneity and robustness. Eighth, our quantitative synthesis was limited to percentage-based outcomes. Continuous and ordinal outcomes were summarized narratively because heterogeneous measures and incomplete reporting precluded separate meta-analysis; their non-inclusion may have affected the completeness of the evidence base or pooled estimates. Finally, there is a high heterogeneity in our dataset, revealing large variability in effect sizes across studies, which may compromise the reliability of the pooled estimates, warranting cautious interpretation of the findings.

## Conclusions

This review suggests that implementation strategies may support modest improvements in implementing DHTs, but this finding was not robust after excluding high-risk studies. More reliable data is needed, given the high risk of bias and the overall effectiveness of strategies varied widely, with limited impact observed on service and patient outcomes, possibly reflecting the complexity of translating implementation success into broader health gains. High heterogeneity of the included studies highlights the importance of tailoring implementation strategies to specific contexts, such as disease types and healthcare providers’ behaviors. Because all included studies were conducted in high-income countries, the findings primarily apply to relatively well-resourced settings. The detailed and comprehensive nature of this study can serve as a reference for policy-makers and clinical practitioners when designing strategies to enhance the implementation of DHTs in managing chronic conditions.

## Supplementary Information

Below is the link to the electronic supplementary material.

Supplementary Materials

## Data Availability

The anonymized datasets generated for this study are available on request from the corresponding authors.

## References

[1] Wang M, Huang K, Li X, Zhao X, Downey L, Hassounah S, et al. Health workers’ adoption of digital health technology in low- and middle-income countries: a systematic review and meta-analysis. Bull World Health Organ. 2025;103(2):126–f35. 10.2471/BLT.24.292157 PMC11774224 39882495

[2] Moschonis G, Siopis G, Jung J, Eweka E, Willems R, Kwasnicka D, et al. Effectiveness, reach, uptake, and feasibility of digital health interventions for adults with type 2 diabetes: a systematic review and meta-analysis of randomised controlled trials. Lancet Digit Health. 2023;5(3):e125–43. 10.1016/S2589-7500(22)00233-3 36828606

[3] Sun S, Simonsson O, McGarvey S, Torous J, Goldberg SB. Mobile phone interventions to improve health outcomes among patients with chronic diseases: an umbrella review and evidence synthesis from 34 meta-analyses. Lancet Digit Health. 2024;6(11):e857–70. 10.1016/S2589-7500(24)00119-5 PMC11534496 39332937

[4] Abraham J, Meng A, Tripathy S, Kitsiou S, Kannampallil T. Effect of health information technology (HIT)-based discharge transition interventions on patient readmissions and emergency room visits: a systematic review. J Am Med Inform Assoc. 2022;29(4):735–48. 10.1093/jamia/ocac013 PMC8922181 35167689

[5] Borges do Nascimento IJ, Abdulazeem HM, Vasanthan LT, Martinez EZ, Zucoloto ML, Østengaard L, et al. The global effect of digital health technologies on health workers’ competencies and health workplace: an umbrella review of systematic reviews and lexical-based and sentence-based meta-analysis. Lancet Digit Health. 2023;5(8):e534–44. 10.1016/S2589-7500(23)00092-4 PMC10397356 37507197

[6] World Health Organization. Global strategy on digital health 2020–2025. Geneva: World Health Organization; 2021.

[7] Gordon WJ, Landman A, Zhang H, Bates DW. Beyond validation: getting health apps into clinical practice. npj Digit Med. 2020;3(1):14. 10.1038/s41746-019-0212-z PMC6997363 32047860

[8] Borges do Nascimento IJ, Abdulazeem H, Vasanthan LT, Martinez EZ, Zucoloto ML, Østengaard L, et al. Barriers and facilitators to utilizing digital health technologies by healthcare professionals. npj Digit Med. 2023;6(1):161. 10.1038/s41746-023-00899-4 PMC10507089 37723240

[9] Proctor EK, Powell BJ, McMillen JC. Implementation strategies: recommendations for specifying and reporting. Implement Sci. 2013;8:139. 10.1186/1748-5908-8-139 PMC3882890 24289295

[10] Powell BJ, Waltz TJ, Chinman MJ, Damschroder LJ, Smith JL, Matthieu MM, et al. A refined compilation of implementation strategies: results from the Expert Recommendations for Implementing Change (ERIC) project. Implement Sci. 2015;10(1):21. 10.1186/s13012-015-0209-1 PMC4328074 25889199

[11] Waltz TJ, Powell BJ, Matthieu MM, Damschroder LJ, Chinman MJ, Smith JL, et al. Use of concept mapping to characterize relationships among implementation strategies and assess their feasibility and importance: results from the Expert Recommendations for Implementing Change (ERIC) study. Implement Sci. 2015;10(1):109. 10.1186/s13012-015-0295-0 PMC4527340 26249843

[12] Li RC, Asch SM, Shah NH. Developing a delivery science for artificial intelligence in healthcare. npj Digit Med. 2020;3(1):107. 10.1038/s41746-020-00318-y PMC7443141 32885053

[13] Ivers N, Jamtvedt G, Flottorp S, Young JM, Odgaard-Jensen J, French SD, et al. Audit and feedback: effects on professional practice and healthcare outcomes. Cochrane Database Syst Rev. 2012;2012(6):Cd000259. 10.1002/14651858.CD000259.pub3 PMC11338587 22696318

[14] Fontaine G, Vinette B, Weight C, Maheu-Cadotte M-A, Lavallée A, Deschênes M-F, et al. Effects of implementation strategies on nursing practice and patient outcomes: a comprehensive systematic review and meta-analysis. Implement Sci. 2024;19(1):68. 10.1186/s13012-024-01398-0 PMC11443951 39350295

[15] Rowe AK, Rowe SY, Peters DH, Holloway KA, Chalker J, Ross-Degnan D. Effectiveness of strategies to improve health-care provider practices in low-income and middle-income countries: a systematic review. Lancet Glob Health. 2018;6(11):e1163–75. 10.1016/S2214-109X(18)30398-X PMC6185992 30309799

[16] Appleton R, Barnett P, Vera San Juan N, Tuudah E, Lyons N, Parker J, et al. Implementation strategies for telemental health: a systematic review. BMC Health Serv Res. 2023;23(1):78. 10.1186/s12913-022-08993-1 PMC9873395 36694164

[17] Ashcraft LE, Goodrich DE, Hero J, Phares A, Bachrach RL, Quinn DA, et al. A systematic review of experimentally tested implementation strategies across health and human service settings: evidence from 2010–2022. Implement Sci. 2024;19(1):43. 10.1186/s13012-024-01369-5 PMC11194895 38915102

[18] Page MJ, McKenzie JE, Bossuyt PM, Boutron I, Hoffmann TC, Mulrow CD, et al. The PRISMA 2020 statement: an updated guideline for reporting systematic reviews. BMJ. 2021;372:n71. 10.1136/bmj.n71 PMC8005924 33782057

[19] Proctor E, Silmere H, Raghavan R, Hovmand P, Aarons G, Bunger A, et al. Outcomes for implementation research: conceptual distinctions, measurement challenges, and research agenda. Adm Policy Ment Health. 2011;38(2):65–76. 10.1007/s10488-010-0319-7 PMC3068522 20957426

[20] Higgins JPT, Thomas J, Chandler J, Cumpston M, Li T, Page MJ, et al. Cochrane handbook for systematic reviews of interventions. wiley; 2019. 10.1002/14651858.ED000142 PMC10284251 31643080

[21] Fontaine G, Cossette S, Maheu-Cadotte MA, Deschênes MF, Rouleau G, Lavallée A, et al. Effect of implementation interventions on nurses’ behaviour in clinical practice: a systematic review, meta-analysis and meta-regression protocol. Syst Rev. 2019;8(1):305. 10.1186/s13643-019-1227-x PMC6896305 31806051

[22] Egger M, Davey Smith G, Schneider M, Minder C. Bias in meta-analysis detected by a simple, graphical test. BMJ. 1997;315(7109):629–34. 10.1136/bmj.315.7109.629 PMC2127453 9310563

[23] Guyatt G, Oxman AD, Akl EA, Kunz R, Vist G, Brozek J, et al. GRADE guidelines: 1. Introduction-GRADE evidence profiles and summary of findings tables. J Clin Epidemiol. 2011;64(4):383–94. 10.1016/j.jclinepi.2010.04.026 21195583

[24] Gordon HS, Pugach O, Solanki P, Gopal RK. A brief pre-visit educational video improved patient engagement after telehealth visits; results from a randomized controlled trial. PEC Innov. 2022;1:100080. 10.1016/j.pecinn.2022.100080 PMC10194152 37213724

[25] Roumie CL, Elasy TA, Greevy R, Griffin MR, Liu X, Stone WJ, et al. Improving blood pressure control through provider education, provider alerts, and patient education: a cluster randomized trial. Ann Intern Med. 2006;145(3):165–75. 10.7326/0003-4819-145-3-200608010-00004 16880458

[26] Feldstein A, Elmer PJ, Smith DH, Herson M, Orwoll E, Chen C, et al. Electronic medical record reminder improves osteoporosis management after a fracture: a randomized, controlled trial. J Am Geriatr Soc. 2006;54(3):450–7. 10.1111/j.1532-5415.2005.00618.x 16551312

[27] Heiman H, Bates DW, Fairchild D, Shaykevich S, Lehmann LS. Improving completion of advance directives in the primary care setting: a randomized controlled trial. Am J Med. 2004;117(5):318–24. 10.1016/j.amjmed.2004.03.027 15336581

[28] Persell SD, Denecke-Dattalo TA, Dunham DP, Baker DW. Patient-directed intervention versus clinician reminders alone to improve aspirin use in diabetes: a cluster randomized trial. Jt Comm J Qual Patient Saf. 2008;34(2):98–105. 10.1016/s1553-7250(08)34012-4 18351194

[29] Kenealy T, Arroll B, Petrie KJ. Patients and computers as reminders to screen for diabetes in family practice. Randomized-controlled trial. J Gen Intern Med. 2005;20(10):916–21. 10.1111/j.1525-1497.2005.0197.x PMC1490216 16191138

[30] Stamenova V, Nguyen M, Onabajo N, Merritt R, Sutakovic O, Mossman K, et al. Mailed Letter Versus Phone Call to Increase Diabetic-Related Retinopathy Screening Engagement by Patients in a Team-Based Primary Care Practice: Prospective, Single-Masked, Randomized Trial. J Med Internet Res. 2023;25:e37867. 10.2196/37867 PMC9878360 36630160

[31] van Wyk JT, van Wijk MA, Sturkenboom MC, Mosseveld M, Moorman PW, van der Lei J. Electronic alerts versus on-demand decision support to improve dyslipidemia treatment: a cluster randomized controlled trial. Circulation. 2008;117(3):371–8. 10.1161/CIRCULATIONAHA.107.697201 18172036

[32] Rosenbloom ST, Geissbuhler AJ, Dupont WD, Giuse DA, Talbert DA, Tierney WM, et al. Effect of CPOE User Interface Design on User-Initiated Access to Educational and Patient Information during Clinical Care. J Am Med Inform Assoc. 2005;12(4):458–73. 10.1197/jamia.M1627 PMC1174891 15802487

[33] Manns B, Tonelli M, Culleton B, Faris P, McLaughlin K, Chin R, et al. A cluster randomized trial of an enhanced eGFR prompt in chronic kidney disease. Clin J Am Soc Nephrol. 2012;7(4):565–72. 10.2215/CJN.12391211 PMC3315340 22344504

[34] Loo TS, Davis RB, Lipsitz LA, Irish J, Bates CK, Agarwal K, et al. Electronic medical record reminders and panel management to improve primary care of elderly patients. Arch Intern Med. 2011;171(17):1552–8. 10.1001/archinternmed.2011.394 21949163

[35] Subramanian U, Fihn SD, Weinberger M, Plue L, Smith FE, Udris EM, et al. A controlled trial of including symptom data in computer-based care suggestions for managing patients with chronic heart failure. Am J Med. 2004;116(6):375–84. 10.1016/j.amjmed.2003.11.021 15006586

[36] Blecker S, Austrian JS, Horwitz LI, Kuperman G, Shelley D, Ferrauiola M, et al. Interrupting providers with clinical decision support to improve care for heart failure. Int J Med Inf. 2019;131:103956. 10.1016/j.ijmedinf.2019.103956 PMC6994190 31525580

[37] Estrada CA, Safford MM, Salanitro AH, Houston TK, Curry W, Williams JH, et al. A web-based diabetes intervention for physician: a cluster-randomized effectiveness trial. Int J Qual Health Care. 2011;23(6):682–9. 10.1093/intqhc/mzr053 PMC3247785 21831967

[38] Bailey FA, Williams BR, Goode PS, Kennedy RE, Redden DT, Kvale E, et al. Comparison of Two Methods for Implementing Comfort Care Order Sets in the Inpatient Setting: a Cluster Randomized Trial. J Gen Intern Med. 2021;36(7):1928–36. 10.1007/s11606-020-06482-x PMC8298677 33547573

[39] Filardo G, Nicewander D, Herrin J, Edwards J, Galimbertti P, Tietze M, et al. A hospital-randomized controlled trial of a formal quality improvement educational program in rural and small community Texas hospitals: one year results. Int J Qual Health Care. 2009;21(4):225–32. 10.1093/intqhc/mzp019 19395710

[40] Gil-Guillén V, Hermida E, Pita-Fernandez S, Palazon-Bru A, Durazo-Arvizu R, Pallares-Carratala V, et al. A cardiovascular educational intervention for primary care professionals in Spain: positive impact in a quasi-experimental study. Br J Gen Pract. 2015;65(630):e32–40. 10.3399/bjgp15X683137 PMC4276005 25548314

[41] Sarna L, Bialous SA, Ong MK, Wells M, Kotlerman J. Increasing nursing referral to telephone quitlines for smoking cessation using a web-based program. Nurs Res. 2012;61(6):433–40. 10.1097/NNR.0b013e3182707237 23073265

[42] Simon SR, Smith DH, Feldstein AC, Perrin N, Yang X, Zhou Y, et al. Computerized prescribing alerts and group academic detailing to reduce the use of potentially inappropriate medications in older people. J Am Geriatr Soc. 2006;54(6):963–8. 10.1111/j.1532-5415.2006.00734.x 16776793

[43] Peels DA, Boekhout JM, van Nassau F, Lechner L, Bolman CAW, Berendsen BAJ. Promoting the implementation of a computer-tailored physical activity intervention: development and feasibility testing of an implementation intervention. Implement Sci Commun. 2024;5(1):90. 10.1186/s43058-024-00622-8 PMC11331651 39160641

[44] Leung S, Zheng WY, Sandhu A, Day R, Li L, Baysari M. Feedback and Training to Improve Use of an Electronic Prescribing System: A Randomised Controlled Trial. Stud Health Technol Inf. 2017;239:63–9. 28756438

[45] Learmonth YC, Mavropalias G, Wansbrough K. Evaluation of a theoretical and experiential training programme for allied healthcare providers to prescribe exercise among persons with multiple sclerosis: a co-designed effectiveness-implementation study. J Clin Med. 2025;14(18):6625. 10.3390/jcm14186625 PMC12470950 41010827

[46] Leonard C, Carey E, Holstein A, Ho PM, Heckman JT. Cluster-randomized implementation trial of two facilitation strategies to implement a novel information and communications technology at the Veterans Health Administration. Implement Sci. 2024;19(1):1. 10.1186/s13012-023-01329-5 PMC10759444 38166974

[47] Alotaibi N, Wilson CB, Traynor M. Enhancing digital readiness and capability in healthcare: a systematic review of interventions, barriers, and facilitators. BMC Health Serv Res. 2025;25(1):500. 10.1186/s12913-025-12663-3 PMC11969766 40186200

[48] Ajzen I. The theory of planned behavior. Organ Behav Hum Decis Process. 1991;50(2):179–211.

[49] Terrana A, Viglione C, Rhee K, Rabin B, Godino J, Aarons GA, et al. The core functions and forms paradigm throughout EPIS: designing and implementing an evidence-based practice with function fidelity. Front Health Serv. 2023;3:1281690. 10.3389/frhs.2023.1281690 PMC10826509 38292916

[50] Miller CJ, Barnett ML, Baumann AA, Gutner CA, Wiltsey-Stirman S. The FRAME-IS: a framework for documenting modifications to implementation strategies in healthcare. Implement Sci. 2021;16(1):36. 10.1186/s13012-021-01105-3 PMC8024675 33827716

[51] Parast L, Doyle B, Damberg CL, Shetty K, Ganz DA, Wenger NS, et al. Challenges in assessing the process-outcome link in practice. J Gen Intern Med. 2015;30(3):359–64. 10.1007/s11606-014-3150-0 PMC4351283 25564435

[52] Breneol S, Curran JA, Marten R, Minocha K, Johnson C, Wong H, et al. Strategies to adapt and implement health system guidelines and recommendations: a scoping review. Health Res Policy Syst. 2022;20(1):64. 10.1186/s12961-022-00865-8 PMC9202131 35706039

[53] Powell BJ, Beidas RS, Lewis CC, Aarons GA, McMillen JC, Proctor EK, et al. Methods to Improve the Selection and Tailoring of Implementation Strategies. J Behav Health Serv Res. 2017;44(2):177–94. 10.1007/s11414-015-9475-6 PMC4761530 26289563

[54] Michie S, van Stralen MM, West R. The behaviour change wheel: a new method for characterising and designing behaviour change interventions. Implement Sci. 2011;6:42. 10.1186/1748-5908-6-42 PMC3096582 21513547

[55] Collins LM, Murphy SA, Strecher V. The multiphase optimization strategy (MOST) and the sequential multiple assignment randomized trial (SMART): new methods for more potent eHealth interventions. Am J Prev Med. 2007;32(5 Suppl):S112–8. 10.1016/j.amepre.2007.01.022 PMC2062525 17466815

