# Supplementary material for: Effectiveness of implementation strategies for digital health implementation in chronic disease care: a systematic review and network meta-analysis
Source: Implement Sci. 2026 Aug 13;21:65. doi: 10.1186/s13012-026-01533-z (PMC13753195; doi:10.1186/s13012-026-01533-z)
Supplement: Supplementary file 1 [file 13012_2026_1533_MOESM1_ESM.docx]

**Effectiveness of Implementation Strategies for Digital Health Implementation in Chronic Disease Care: A Systematic Review and Network Meta-analysis**

**Supplementary Materials**

**Table of Contents**

Supplementary Material 1: PRISMA checklist

Supplementary Material 2: Search strings

Supplementary Material 3: Updated search

Supplementary Material 4: Inclusion and exclusion criteria

Supplementary Material 5: Definition of the strategy category and sub-category

Supplementary Material 6: Methodological details

Supplementary Material 7: List of excluded studies with reasons for exclusion

Supplementary Material 8: Characteristics of included studies

Supplementary Material 9: Risk of bias

Supplementary Material 10: GRADE scoring process

Supplementary Material 11: Additional results of network meta-analysis

Supplementary Material 12: Secondary outcome (service and patient outcomes) results

Supplementary Material 13: Effects of implementation strategies in included studies not eligible for meta-analysis

Supplementary Material 14: Meta-regression

Supplementary Material 15: Subgroup analysis

Supplementary Material 16: Publication bias

Supplementary Material 17: Sensitivity Analysis

**Supplementary Material 1: PRISMA checklist**

| **Section and Topic** | **Item #** | **Checklist item** | **Location where item is reported** |
| --- | --- | --- | --- |
| **TITLE** | | |  |
| Title | 1 | Identify the report as a systematic review. | Page 1 |
| **ABSTRACT** | | |  |
| Abstract | 2 | See the PRISMA 2020 for Abstracts checklist. | Page 1 |
| **INTRODUCTION** | | |  |
| Rationale | 3 | Describe the rationale for the review in the context of existing knowledge. | Page 2 |
| Objectives | 4 | Provide an explicit statement of the objective(s) or question(s) the review addresses. | Page 2-3 |
| **METHODS** | | |  |
| Eligibility criteria | 5 | Specify the inclusion and exclusion criteria for the review and how studies were grouped for the syntheses. | Page 3, appx 3 |
| Information sources | 6 | Specify all databases, registers, websites, organisations, reference lists and other sources searched or consulted to identify studies. Specify the date when each source was last searched or consulted. | Page 3, figure 1 |
| Search strategy | 7 | Present the full search strategies for all databases, registers and websites, including any filters and limits used. | Appx 2 |
| Selection process | 8 | Specify the methods used to decide whether a study met the inclusion criteria of the review, including how many reviewers screened each record and each report retrieved, whether they worked independently, and if applicable, details of automation tools used in the process. | Page 3 |
| Data collection process | 9 | Specify the methods used to collect data from reports, including how many reviewers collected data from each report, whether they worked independently, any processes for obtaining or confirming data from study investigators, and if applicable, details of automation tools used in the process. | Page 4 |
| Data items | 10a | List and define all outcomes for which data were sought. Specify whether all results that were compatible with each outcome domain in each study were sought (e.g. for all measures, time points, analyses), and if not, the methods used to decide which results to collect. | Page 3-4 |
|  | 10b | List and define all other variables for which data were sought (e.g. participant and intervention characteristics, funding sources). Describe any assumptions made about any missing or unclear information. | Page 3-4 |
| Study risk of bias assessment | 11 | Specify the methods used to assess risk of bias in the included studies, including details of the tool(s) used, how many reviewers assessed each study and whether they worked independently, and if applicable, details of automation tools used in the process. | Page 5-6 |
| Effect measures | 12 | Specify for each outcome the effect measure(s) (e.g. risk ratio, mean difference) used in the synthesis or presentation of results. | Page 4-5, appx 5 |
| Synthesis methods | 13a | Describe the processes used to decide which studies were eligible for each synthesis (e.g. tabulating the study intervention characteristics and comparing against the planned groups for each synthesis (item #5)). | Page 4 |
|  | 13b | Describe any methods required to prepare the data for presentation or synthesis, such as handling of missing summary statistics, or data conversions. | Appx 5 |
|  | 13c | Describe any methods used to tabulate or visually display results of individual studies and syntheses. | Page 4-5 |
|  | 13d | Describe any methods used to synthesize results and provide a rationale for the choice(s). If meta-analysis was performed, describe the model(s), method(s) to identify the presence and extent of statistical heterogeneity, and software package(s) used. | Page 4-5 |
|  | 13e | Describe any methods used to explore possible causes of heterogeneity among study results (e.g. subgroup analysis, meta-regression). | Page 5 |
|  | 13f | Describe any sensitivity analyses conducted to assess robustness of the synthesized results. | Page 6 |
| Reporting bias assessment | 14 | Describe any methods used to assess risk of bias due to missing results in a synthesis (arising from reporting biases). | Page 5 |
| Certainty assessment | 15 | Describe any methods used to assess certainty (or confidence) in the body of evidence for an outcome. | Page 5-6 |
| **RESULTS** | | |  |
| Study selection | 16a | Describe the results of the search and selection process, from the number of records identified in the search to the number of studies included in the review, ideally using a flow diagram. | Page 6, Figure 1 |
|  | 16b | Cite studies that might appear to meet the inclusion criteria, but which were excluded, and explain why they were excluded. | Appx 6 |
| Study characteristics | 17 | Cite each included study and present its characteristics. | Page 6-7 |
| Risk of bias in studies | 18 | Present assessments of risk of bias for each included study. | Page 7, appx 8 |
| Results of individual studies | 19 | For all outcomes, present, for each study: (a) summary statistics for each group (where appropriate) and (b) an effect estimate and its precision (e.g. confidence/credible interval), ideally using structured tables or plots. | Table 1 |
| Results of syntheses | 20a | For each synthesis, briefly summarise the characteristics and risk of bias among contributing studies. | Page 7-9, appx10-15 |
|  | 20b | Present results of all statistical syntheses conducted. If meta-analysis was done, present for each the summary estimate and its precision (e.g. confidence/credible interval) and measures of statistical heterogeneity. If comparing groups, describe the direction of the effect. | Page 7-10, appx10-15 |
|  | 20c | Present results of all investigations of possible causes of heterogeneity among study results. | Page 9, appx 14-15 |
|  | 20d | Present results of all sensitivity analyses conducted to assess the robustness of the synthesized results. | Page 10; Appx 17 |
| Reporting biases | 21 | Present assessments of risk of bias due to missing results (arising from reporting biases) for each synthesis assessed. | Appx 8 |
| Certainty of evidence | 22 | Present assessments of certainty (or confidence) in the body of evidence for each outcome assessed. | Table 1, Appx 9 |
| **DISCUSSION** | | |  |
| Discussion | 23a | Provide a general interpretation of the results in the context of other evidence. | Page 10-13 |
|  | 23b | Discuss any limitations of the evidence included in the review. | Page 13 |
|  | 23c | Discuss any limitations of the review processes used. | Page 13 |
|  | 23d | Discuss implications of the results for practice, policy, and future research. | Page 13 |
| **OTHER INFORMATION** | | |  |
| Registration and protocol | 24a | Provide registration information for the review, including register name and registration number, or state that the review was not registered. | Page 3 |
|  | 24b | Indicate where the review protocol can be accessed, or state that a protocol was not prepared. | Page 3 |
|  | 24c | Describe and explain any amendments to information provided at registration or in the protocol. | NA |
| Support | 25 | Describe sources of financial or non-financial support for the review, and the role of the funders or sponsors in the review. | NA |
| Competing interests | 26 | Declare any competing interests of review authors. | Page 15 |
| Availability of data, code and other materials | 27 | Report which of the following are publicly available and where they can be found: template data collection forms; data extracted from included studies; data used for all analyses; analytic code; any other materials used in the review. | Page 15 |

**Supplementary Material 2: Search strings**

**Search date:** July 4^th^, 2024

**Database:** MEDLINE (Ovid), Embase (Ovid), PsycINFO, Scopus

**MEDLINE, Embase, and PsycINFO** 1946 to 2024 July 4^th^

|  |  | **Embase** | **MEDLINE** | **PsycINFO** |
| --- | --- | --- | --- | --- |
| 1 | exp Telemedicine/ | 74959 | 46752 | 14695 |
| 2 | ((Digital or mobile or smart) adj3 health).ti,ab. | 18005 | 10847 | 3029 |
| 3 | (telehealth or telemedicine or telemonitoring).ti,ab. | 43586 | 24783 | 5942 |
| 4 | Mobile Applications/ | 21970 | 12265 | 2885 |
| 5 | exp Internet/ | 132771 | 99704 | 34097 |
| 6 | exp Cell Phone/ | 50416 | 23319 | 8125 |
| 7 | exp Computers, Handheld/ | 1864 | 13682 |  |
| 8 | Medical Informatics Applications/ | 23724 | 2552 |  |
| 9 | Therapy, Computer-Assisted/ | 4627 | 6980 |  |
| 10 | (app or apps).ti,ab. | 62622 | 34929 | 12031 |
| 11 | (online or internet or web* or digital* or tele* or google).ti. | 252856 | 174228 | 84788 |
| 12 | ((online or internet or web* or digital*) adj3 (based or application* or intervention* or program* or therap*)).ab. | 115519 | 65710 | 35901 |
| 13 | (phone* or telephone* or smartphone* or cellphone* or smartwatch*).ti. | 33491 | 22606 | 11975 |
| 14 | ((phone* or telephone* or smartphone* or cellphone* or smartwatch*) adj3 (based or application* or intervention* or program* or therap*)).ab. | 23870 | 14038 | 6362 |
| 15 | (mobile health or mhealth or m-health or ehealth or e-health or emental or e-mental).ti. | 9934 | 6609 | 2008 |
| 16 | ((mobile health or mhealth or m-health or ehealth or e-health or emental or e-mental) adj3 (based or application* or intervention* or program* or therap*)).ab. | 6930 | 4466 | 1670 |
| 17 | (mobile* adj3 (based or application* or intervention* or device* or technolog*)).ti,ab. | 28581 | 15880 | 9327 |
| 18 | (Connected Devices or Smart Devices or Digital Assistant).ti,ab. | 2366 | 1260 | 491 |
| 19 | exp Medical Informatics/ or exp Medical Records Systems, Computerized/ | 26662 | 524467 |  |
| 20 | ((Health or medical) adj3 (record* or informati* or data)).ti,ab. | 496240 | 272240 | 61970 |
| 21 | (digital record* or personalized medicine or personalised medicine or interoperability).ti,ab. | 30622 | 17073 | 1794 |
| 22 | exp Artificial Intelligence/ | 97134 | 190339 | 68378 |
| 23 | ((Artificial or machine or deep or hierarchical or ambient or comput*) adj3 (intelligence or learning)).ti,ab. | 200417 | 106691 | 27436 |
| 24 | ((Computer or automated) adj3 reasoning).ti,ab. | 201 | 153 | 111 |
| 25 | (Knowledge adj3 (acquisition or representation*)).ti,ab. | 7277 | 4850 | 7431 |
| 26 | natural language processing.ti,ab. | 8994 | 5032 | 1815 |
| 27 | (AI or NLP).ti,ab. | 74111 | 40820 | 8234 |
| 28 | exp Social Media/ | 53280 | 16960 | 24904 |
| 29 | (social media or social network or Facebook or twitter or youtube or Instagram or flickr or Linkedin or blog* or on-line communit* or online communit* or wiki* or big data or open data or data mining or cloud or bluetooth or wearable* or wireless technology).ti,ab. | 157479 | 86968 | 57954 |
| 30 | 1 or 2 or 3 or 4 or 5 or 6 or 7 or 8 or 9 or 10 or 11 or 12 or 13 or 14 or 15 or 16 or 17 or 18 or 19 or 20 or 21 or 22 or 23 or 24 or 25 or 26 or 27 or 28 or 29 | 1459704 | 1285573 | 332713 |
| 31 | exp health personnel/ | 2034718 | 628104 | 198718 |
| 32 | exp Physicians/ | 997727 | 183491 | 50127 |
| 33 | (physician* or doctor*).ti,ab. | 863049 | 520429 | 110072 |
| 34 | exp Nurses/ | 223089 | 100093 | 39751 |
| 35 | (nurse* or nursing).ti,ab. | 620520 | 479346 | 118157 |
| 36 | (clinical officer* or medical officer*).ti,ab. | 5248 | 3558 | 524 |
| 37 | Nurses' Aides/ | 4949 | 4430 |  |
| 38 | (health auxiliar* or health assistant*).ti,ab. | 449 | 342 | 62 |
| 39 | Pharmacists/ | 94714 | 22425 | 2005 |
| 40 | pharmacist*.ti,ab. | 94516 | 35683 | 4042 |
| 41 | Community Health Workers/ | 9514 | 6756 |  |
| 42 | community health worker*.ti,ab. | 7872 | 5441 | 1332 |
| 43 | ((Health* or medical) adj3 (personnel or professional* or provider* or worker* or practitioner* or aide*)).ti,ab. | 480688 | 309303 | 123322 |
| 44 | 31 or 32 or 33 or 34 or 35 or 36 or 37 or 38 or 39 or 40 or 41 or 42 or 43 | 2972798 | 1516711 | 409617 |
| 45 | randomized controlled trial.pt. |  | 609369 |  |
| 46 | controlled clinical trial.pt. |  | 95550 |  |
| 47 | multicenter study.pt. |  | 343678 |  |
| 48 | (randomis* or randomiz* or randomly allocat* or random allocat*).ti,ab. | 1240589 | 748754 | 115289 |
| 49 | groups.ab. | 3701944 | 2230594 | 574520 |
| 50 | (trial or multicenter or multi center or multicentre or multi centre).ti. | 517955 | 318879 | 42829 |
| 51 | (intervention* or controlled or control group or compare or compared or (before adj5 after) or (pre adj5 post) or pretest or pre test or posttest or post test or quasiexperiment* or quasi experiment* or evaluat* or effect or impact or time series or time point? or repeated measur*).ti,ab. | 16826460 | 10819522 | 2253378 |
| 52 | 45 or 46 or 47 or 48 or 49 or 50 or 51 | 17936758 | 11690353 | 2508188 |
| 53 | exp Education, Continuing/ | 33669 | 62862 | 1978 |
| 54 | (education$ adj2 (program$ or intervention? or meeting? or session? or strateg$ or workshop? or visit?)).tw. | 125133 | 75895 | 59751 |
| 55 | *Pamphlets/ | 44895 | 1898 |  |
| 56 | (behavio?r$ adj2 intervention?).tw. | 26612 | 17485 | 21594 |
| 57 | (leaflet? or booklet? or poster or posters).tw. | 74928 | 32730 | 6464 |
| 58 | ((written or printed or oral) adj information).tw. | 4645 | 2359 | 846 |
| 59 | (information$ adj2 campaign?).tw. | 2034 | 1397 | 524 |
| 60 | (education$ adj1 (method? or material?)).tw. | 20360 | 8907 | 4247 |
| 61 | outreach.tw. | 27037 | 15845 | 9443 |
| 62 | ((opinion or education$ or influential) adj1 leader?).tw. | 3102 | 1944 | 3953 |
| 63 | facilitator?.tw. | 51265 | 34124 | 20971 |
| 64 | academic detailing.tw. | 923 | 567 | 122 |
| 65 | consensus conference?.tw. | 8726 | 5754 | 471 |
| 66 | practice guideline?.tw. | 47952 | 29195 | 5515 |
| 67 | (guideline? adj2 (introduc$ or issu$ or impact or effect? or disseminat$ or distribut$)).tw. | 12116 | 5781 | 1096 |
| 68 | ((effect? or impact or evaluat$ or introduc$ or compar$) adj2 training program$).tw. | 1637 | 1019 | 884 |
| 69 | *reminder systems/ | 1207 | 2285 |  |
| 70 | reminder?.tw. | 26378 | 13910 | 7489 |
| 71 | (recall adj2 system$).tw. | 777 | 553 | 132 |
| 72 | (prompter? or prompting).tw. | 21240 | 10704 | 5448 |
| 73 | algorith?.tw. | 348202 | 189918 | 27502 |
| 74 | *feedback/ or feedback.tw. | 238732 | 159934 | 82668 |
| 75 | (feedback adj1 (loop? or control? or regula$ or mechanism? or inhib$ or system? or circuit? or sensory or visual or audi$)).tw. | 71300 | 55623 | 12835 |
| 76 | 74 not 75 | 167432 | 104311 | 69833 |
| 77 | chart review$.tw. | 118761 | 47142 |  |
| 78 | ((effect? or impact or records or chart?) adj2 audit).tw. | 2340 | 1174 | 261 |
| 79 | *patient education/ | 29921 | 40825 |  |
| 80 | marketing.tw. | 46087 | 27770 | 34423 |
| 81 | ((effect? or impact or evaluat? or introduc$ or compara$) adj2 (prevent$ program$ or screening program$)).tw. | 949 | 699 |  |
| 82 | ((introduc$ or impact or effect? or implement$ or computer$) adj2 protocol?).tw. | 9419 | 5879 |  |
| 83 | (computer$ adj2 (dosage or dosing or diagnosis or therapy or decision?)).tw. | 8751 | 7176 |  |
| 84 | *physician's practice patterns/ | 48724 | 41237 | 15927 |
| 85 | or/53-73,76-84 | 1193449 | 600220 | 288519 |
| 86 | (chronic care or chronic condition or chronic health or chronic disease or diabetes or T2DM *or* heart failure or CHF or chronic obstructive pulmonary disease or COPD or emphysema or COAD or *c*ardiovascular disease).ti,ab. | 1730962 | 968412 | 60460 |
| 87 | 30 and 44 and 52 and 85 and 86 | 4226 | 1534 | 323 |

**Scopus: 3199**

1. **DHT:**

"digital" OR tele* OR "mhealth" OR "m-health" OR "ehealth" OR "e-health" OR "emental" OR "e-mental" OR "internet" OR "mobile phone" OR "computer assisted therapy" OR "connected devices" OR "smart devices" OR "online" OR web* OR "google" OR phone* OR smartphone* OR cellphone* OR smartwatch* OR "mobile health" OR "smart health" OR "mobile based" OR "mobile application" OR "mobile applications" OR "mobile intervention" OR "mobile interventions" OR "mobile device" OR "mobile devices" OR "mobile technology" OR "mobile technologies" OR "app" OR "apps" OR "social media" OR "social network" OR "Facebook" OR "twitter" OR "youtube" OR "Instagram" OR "flickr" OR "Linkedin" OR blog* OR "on-line community" OR "on-line communities" OR "online community" OR "online communities" OR wiki* OR "big data" OR "open data" OR "data mining" OR cloud OR bluetooth OR wearable* OR "wireless technology" OR "health record" OR "health records" OR "medical record" OR "medical records" OR "health information" OR "medical information" OR "health data" OR "medical data" OR "personalized medicine" OR "interoperability" OR "natural language processing" OR "AI" OR "NLP" OR "artificial intelligence" OR "machine intelligence" OR "deep intelligence" OR "hierarchical intelligence" OR "ambient intelligence" OR "computer intelligence" OR "artificial learning" OR "machine learning" OR "deep learning" OR "hierarchical learning" OR "ambient learning" OR "computer learning" OR "computer reasoning" OR "automated reasoning" OR "knowledge acquisition" OR "knowledge representation" OR "knowledge representations"

2. **HCPs:**

"health personnel" OR "healthcare personnel" OR "health professional" OR "health professionals" OR "healthcare professional" OR "healthcare professionals" OR "health provider" OR "health providers" OR "healthcare provider" OR "healthcare providers" OR "health worker" OR "health workers" OR "healthcare worker" OR "healthcare workers" OR "health practitioner" OR "health practitioners" OR "healthcare practitioner" OR "healthcare practitioners" OR "health aide" OR "health aides" OR "healthcare aide" OR "healthcare aides" OR "medical personnel" OR "medical professional" OR "medical professionals" OR "medical provider" OR "medical providers" OR "medical worker" OR "medical workers" OR "medical practitioner" OR "medical practitioners" OR "medical aide" OR "medical aides"

3. **Study design:**

(randomis*) OR (randomize*) OR (randomly allocate*) OR (random allocate*) OR (trail) OR (multicenter) OR (multi center) OR (multicentre) OR (multi centre) OR (intervention) OR (controlled) OR ( control group) OR (compare) OR (compared) OR (before W/5 after) OR (pre W/5 post) OR (pretest) OR (pre test) OR (posttest) OR (post test) OR ( quasiexperiment*) OR (quasi experiment*) OR (evaluat*) OR (effect) OR (impact) OR (time series) OR (time point?) OR (repeated measur*)

4. **Implementation strategy:**

(education* AND (program* OR intervention* OR meeting* OR session* OR strateg* OR workshop* OR visit* OR method* OR material*)) OR (behavio* AND intervention*) OR (leaflet* OR booklet* OR poster OR posters) OR ((written OR printed OR oral OR campaign*) AND information) OR outreach OR ((opinion OR education* OR influential) AND leader*) OR facilitator* OR "academic detailing" OR "consensus conference*" OR "practice guideline*" OR (guideline* AND (introduc* OR issu* OR impact OR effect* OR disseminat* OR distribut*)) OR ((effect* OR impact OR evaluat* OR introduc* OR compar*) AND "training program*") OR reminder* OR (recall AND system*) OR prompter* OR prompting OR algorith* OR "chart review*" OR ((effect* OR impact OR records OR chart*) AND audit) OR marketing OR ((effect* OR impact OR evaluat* OR introduc* OR compara*) AND ("prevent* program*" OR "screening program*")) OR ((introduc* OR impact OR effect* OR implement* OR computer*) AND protocol*) OR (computer* AND (dosage OR dosing OR diagnosis OR therapy OR decision*))

5. **Chronic disease:**

"chronic care" OR "chronic condition" OR "chronic health" OR "chronic disease" OR "diabetes" OR "T2DM" OR "heart failure" OR "CHF" OR "chronic obstructive pulmonary disease" OR "COPD" OR "cardiovascular disease" OR "heart disease" OR "heart diseases" OR "COAD" OR "emphysema"

**Supplementary Material 3: Updated Search**

**Search date:** 2026 March 16^th^

**Database:** MEDLINE (Ovid), Embase (Ovid), PsycINFO, Scopus

**MEDLINE, Embase, and PsycINFO** 2024 July to 2026 Current

|  |  | **Embase** | **MEDLINE** | **PsycINFO** |
| --- | --- | --- | --- | --- |
| 1 | exp Telemedicine/ | 18739 | 10491 | 142 |
| 2 | ((Digital or mobile or smart) adj3 health).ti,ab. | 11862 | 6685 | 1551 |
| 3 | (telehealth or telemedicine or telemonitoring).ti,ab. | 13021 | 6411 | 1802 |
| 4 | Mobile Applications/ | 7826 | 4748 | 842 |
| 5 | exp Internet/ | 13718 | 11839 | 1066 |
| 6 | exp Cell Phone/ | 15311 | 4650 | 901 |
| 7 | exp Computers, Handheld/ | 141 | 3585 | - |
| 8 | Medical Informatics Applications/ | 1985 | 4 | - |
| 9 | Therapy, Computer-Assisted/ | 115 | 104 | - |
| 10 | (app or apps).ti,ab. | 18417 | 8964 | 2981 |
| 11 | (online or internet or web* or digital* or tele* or google).ti. | 53272 | 26857 | 13210 |
| 12 | ((online or internet or web* or digital*) adj3 (based or application* or intervention* or program* or therap*)).ab. | 29139 | 15238 | 5799 |
| 13 | (phone* or telephone* or smartphone* or cellphone* or smartwatch*).ti. | 5959 | 3351 | 1347 |
| 14 | ((phone* or telephone* or smartphone* or cellphone* or smartwatch*) adj3 (based or application* or intervention* or program* or therap*)).ab. | 6056 | 3306 | 850 |
| 15 | (mobile health or mhealth or m-health or ehealth or e-health or emental or e-mental).ti. | 2685 | 1682 | 469 |
| 16 | ((mobile health or mhealth or m-health or ehealth or e-health or emental or e-mental) adj3 (based or application* or intervention* or program* or therap*)).ab. | 2590 | 1529 | 447 |
| 17 | (mobile* adj3 (based or application* or intervention* or device* or technolog*)).ti,ab. | 8886 | 4240 | 1453 |
| 18 | (Connected Devices or Smart Devices or Digital Assistant).ti,ab. | 664 | 271 | 72 |
| 19 | exp Medical Informatics/ or exp Medical Records Systems, Computerized/ | 3405 | 44235 |  |
| 20 | ((Health or medical) adj3 (record* or informati* or data)).ti,ab. | 110217 | 49020 | 8168 |
| 21 | (digital record* or personalized medicine or personalised medicine or interoperability).ti,ab. | 10091 | 4975 | 249 |
| 22 | exp Artificial Intelligence/ | 95161 | 88632 | 15975 |
| 23 | ((Artificial or machine or deep or hierarchical or ambient or comput*) adj3 (intelligence or learning)).ti,ab. | 172192 | 82763 | 10308 |
| 24 | ((Computer or automated) adj3 reasoning).ti,ab. | 30 | 11 | 4 |
| 25 | (Knowledge adj3 (acquisition or representation*)).ti,ab. | 1553 | 859 | 553 |
| 26 | natural language processing.ti,ab. | 5492 | 2637 | 759 |
| 27 | (AI or NLP).ti,ab. | 67594 | 27640 | 6166 |
| 28 | exp Social Media/ | 23894 | 5107 | 5484 |
| 29 | (social media or social network or Facebook or twitter or youtube or Instagram or flickr or Linkedin or blog* or on-line communit* or online communit* or wiki* or big data or open data or data mining or cloud or bluetooth or wearable* or wireless technology).ti,ab. | 52365 | 25400 | 11745 |
| 30 | 1 or 2 or 3 or 4 or 5 or 6 or 7 or 8 or 9 or 10 or 11 or 12 or 13 or 14 or 15 or 16 or 17 or 18 or 19 or 20 or 21 or 22 or 23 or 24 or 25 or 26 or 27 or 28 or 29 | 469869 | 243198 | 55011 |
| 31 | exp health personnel/ | 306470 | 53811 | 22089 |
| 32 | exp Physicians/ | 133933 | 15374 | 2185 |
| 33 | (physician* or doctor*).ti,ab. | 97724 | 41248 | 6192 |
| 34 | exp Nurses/ | 29051 | 5665 | 3979 |
| 35 | (nurse* or nursing).ti,ab. | 75263 | 40691 | 9188 |
| 36 | (clinical officer* or medical officer*).ti,ab. | 685 | 277 | 33 |
| 37 | Nurses' Aides/ | 499 | 147 |  |
| 38 | (health auxiliar* or health assistant*).ti,ab. | 106 | 54 | 13 |
| 39 | Pharmacists/ | 14782 | 2652 | 210 |
| 40 | pharmacist*.ti,ab. | 15744 | 4664 | 381 |
| 41 | Community Health Workers/ | 2626 | 787 |  |
| 42 | community health worker*.ti,ab. | 2276 | 1178 | 232 |
| 43 | ((Health* or medical) adj3 (personnel or professional* or provider* or worker* or practitioner* or aide*)).ti,ab. | 102515 | 53971 | 14884 |
| 44 | 31 or 32 or 33 or 34 or 35 or 36 or 37 or 38 or 39 or 40 or 41 or 42 or 43 | 413743 | 142277 | 38404 |
| 45 | randomized controlled trial.pt. |  | 55107 |  |
| 46 | controlled clinical trial.pt. |  | 460 |  |
| 47 | multicenter study.pt. |  | 52325 |  |
| 48 | (randomis* or randomiz* or randomly allocat* or random allocat*).ti,ab. | 245364 | 111189 | 14600 |
| 49 | groups.ab. | 563625 | 265394 | 43745 |
| 50 | (trial or multicenter or multi center or multicentre or multi centre).ti. | 130801 | 57482 | 5446 |
| 51 | (intervention* or controlled or control group or compare or compared or (before adj5 after) or (pre adj5 post) or pretest or pre test or posttest or post test or quasiexperiment* or quasi experiment* or evaluat* or effect or impact or time series or time point? or repeated measur*).ti,ab. | 2585153 | 1258423 | 205910 |
| 52 | 45 or 46 or 47 or 48 or 49 or 50 or 51 | 2715485 | 1328315 | 219690 |
| 53 | exp Education, Continuing/ | 1584 | 759 |  |
| 54 | (education$ adj2 (program$ or intervention? or meeting? or session? or strateg$ or workshop? or visit?)).tw. | 23711 | 11234 | 5608 |
| 55 | *Pamphlets/ | 2281 | 100 |  |
| 56 | (behavio?r$ adj2 intervention?).tw. | 5800 | 3084 | 2223 |
| 57 | (leaflet? or booklet? or poster or posters).tw. | 17170 | 2866 | 391 |
| 58 | ((written or printed or oral) adj information).tw. | 587 | 77 | 46 |
| 59 | (information$ adj2 campaign?).tw. | 238 | 123 | 24 |
| 60 | (education$ adj1 (method? or material?)).tw. | 5796 | 1823 | 419 |
| 61 | outreach.tw. | 6734 | 2977 | 1067 |
| 62 | ((opinion or education$ or influential) adj1 leader?).tw. | 538 | 278 | 781 |
| 63 | facilitator?.tw. | 16631 | 9536 | 3955 |
| 64 | academic detailing.tw. | 85 | 50 | 10 |
| 65 | consensus conference?.tw. | 524 | 288 | 29 |
| 66 | practice guideline?.tw. | 8504 | 4109 | 529 |
| 67 | (guideline? adj2 (introduc$ or issu$ or impact or effect? or disseminat$ or distribut$)).tw. | 1993 | 798 | 62 |
| 68 | ((effect? or impact or evaluat$ or introduc$ or compar$) adj2 training program$).tw. | 262 | 122 | 65 |
| 69 | *reminder systems/ | 108 | 165 |  |
| 70 | reminder?.tw. | 5448 | 1960 | 740 |
| 71 | (recall adj2 system$).tw. | 92 | 35 | 13 |
| 72 | (prompter? or prompting).tw. | 13968 | 5288 | 1042 |
| 73 | algorith?.tw. | 75208 | 30972 | 3116 |
| 74 | *feedback/ or feedback.tw. | 48435 | 22197 | 8678 |
| 75 | (feedback adj1 (loop? or control? or regula$ or mechanism? or inhib$ or system? or circuit? or sensory or visual or audi$)).tw. | 10447 | 5769 | 1110 |
| 76 | 74 not 75 | 48435 | 5769 | 8678 |
| 77 | chart review$.tw. | 19940 | 5835 |  |
| 78 | ((effect? or impact or records or chart?) adj2 audit).tw. | 236 | 92 | 21 |
| 79 | *patient education/ | 2769 | 2359 |  |
| 80 | marketing.tw. | 6772 | 3095 | 3005 |
| 81 | ((effect? or impact or evaluat? or introduc$ or compara$) adj2 (prevent$ program$ or screening program$)).tw. | 122 | 48 |  |
| 82 | ((introduc$ or impact or effect? or implement$ or computer$) adj2 protocol?).tw. | 3165 | 1350 |  |
| 83 | (computer$ adj2 (dosage or dosing or diagnosis or therapy or decision?)).tw. | 1472 | 788 |  |
| 84 | *physician's practice patterns/ | 9312 | 3302 |  |
| 85 | or/53-73,76-84 | 257856 | 107379 | 29742 |
| 86 | (chronic care or chronic condition or chronic health or chronic disease or diabetes or T2DM *or* heart failure or CHF or chronic obstructive pulmonary disease or COPD or emphysema or COAD or *c*ardiovascular disease).ti,ab. | 273988 | 116223 | 5162 |
| 87 | 30 and 44 and 52 and 85 and 86 | 1530 | 451 | 57 |

**Scopus （N=1388）**

1. **DHTs**

"digital" OR tele* OR "mhealth" OR "m-health" OR "ehealth" OR "e-health" OR "emental" OR "e-mental" OR "internet" OR "mobile phone" OR "computer assisted therapy" OR "connected devices" OR "smart devices" OR "online" OR web* OR "google" OR phone* OR smartphone* OR cellphone* OR smartwatch* OR "mobile health" OR "smart health" OR "mobile based" OR "mobile application" OR "mobile applications" OR "mobile intervention" OR "mobile interventions" OR "mobile device" OR "mobile devices" OR "mobile technology" OR "mobile technologies" OR "app" OR "apps" OR "social media" OR "social network" OR "Facebook" OR "twitter" OR "youtube" OR "Instagram" OR "flickr" OR "Linkedin" OR blog* OR "on-line community" OR "on-line communities" OR "online community" OR "online communities" OR wiki* OR "big data" OR "open data" OR "data mining" OR cloud OR bluetooth OR wearable* OR "wireless technology" OR "health record" OR "health records" OR "medical record" OR "medical records" OR "health information" OR "medical information" OR "health data" OR "medical data" OR "personalized medicine" OR "interoperability" OR "natural language processing" OR "AI" OR "NLP" OR "artificial intelligence" OR "machine intelligence" OR "deep intelligence" OR "hierarchical intelligence" OR "ambient intelligence" OR "computer intelligence" OR "artificial learning" OR "machine learning" OR "deep learning" OR "hierarchical learning" OR "ambient learning" OR "computer learning" OR "computer reasoning" OR "automated reasoning" OR "knowledge acquisition" OR "knowledge representation" OR "knowledge representations"

2. **HCPs**

"health personnel" OR "healthcare personnel" OR "health professional" OR "health professionals" OR "healthcare professional" OR "healthcare professionals" OR "health provider" OR "health providers" OR "healthcare provider" OR "healthcare providers" OR "health worker" OR "health workers" OR "healthcare worker" OR "healthcare workers" OR "health practitioner" OR "health practitioners" OR "healthcare practitioner" OR "healthcare practitioners" OR "health aide" OR "health aides" OR "healthcare aide" OR "healthcare aides" OR "medical personnel" OR "medical professional" OR "medical professionals" OR "medical provider" OR "medical providers" OR "medical worker" OR "medical workers" OR "medical practitioner" OR "medical practitioners" OR "medical aide" OR "medical aides"

3. **Study design**

(randomis*) OR (randomize*) OR (randomly allocate*) OR (random allocate*) OR (trail) OR (multicenter) OR (multi center) OR (multicentre) OR (multi centre) OR (intervention) OR (controlled) OR ( control group) OR (compare) OR (compared) OR (before W/5 after) OR (pre W/5 post) OR (pretest) OR (pre test) OR (posttest) OR (post test) OR ( quasiexperiment*) OR (quasi experiment*) OR (evaluat*) OR (effect) OR (impact) OR (time series) OR (time point?) OR (repeated measur*)

4. **Implementation strategy**

(education* AND (program* OR intervention* OR meeting* OR session* OR strateg* OR workshop* OR visit* OR method* OR material*)) OR (behavio* AND intervention*) OR (leaflet* OR booklet* OR poster OR posters) OR ((written OR printed OR oral OR campaign*) AND information) OR outreach OR ((opinion OR education* OR influential) AND leader*) OR facilitator* OR "academic detailing" OR "consensus conference*" OR "practice guideline*" OR (guideline* AND (introduc* OR issu* OR impact OR effect* OR disseminat* OR distribut*)) OR ((effect* OR impact OR evaluat* OR introduc* OR compar*) AND "training program*") OR reminder* OR (recall AND system*) OR prompter* OR prompting OR algorith* OR "chart review*" OR ((effect* OR impact OR records OR chart*) AND audit) OR marketing OR ((effect* OR impact OR evaluat* OR introduc* OR compara*) AND ("prevent* program*" OR "screening program*")) OR ((introduc* OR impact OR effect* OR implement* OR computer*) AND protocol*) OR (computer* AND (dosage OR dosing OR diagnosis OR therapy OR decision*))

5. **Chronic disease**

"chronic care" OR "chronic condition" OR "chronic health" OR "chronic disease" OR "diabetes" OR "T2DM" OR "heart failure" OR "CHF" OR "chronic obstructive pulmonary disease" OR "COPD" OR "cardiovascular disease" OR "heart disease" OR "heart diseases" OR "COAD" OR "emphysema"

**Supplementary Material 4: Inclusion and exclusion criteria**

1) Publication type: peer-reviewed research articles. Not a protocol.
2) Timing and setting: no restrictions
3) Type of health condition: chronic diseases, such as HF, COPD, and diabetes…
4) Type of HCPs: Hospital or health facility-based health worker and community health work
5) The DHTs being implemented should be well defined and described. The possible DHTs types are like: Mobile Health, Telemedicine, Electronic Health Record, CDSS, Clinical reminder, e-Health, Social media, and AI (1).
6) Eligible strategies had to include at least one intervention component that could plausibly affect implementation. The intervention component is classified according to ERIC (2).
7) Study design:
   a. Randomized controlled before-and-after trials
   b. Non-randomized controlled before-and-after trials
   c. Randomized controlled post-intervention only trials
   d. Interrupted time series (ITS) designs with at least 3 data points before and after the intervention, with or without comparison groups
8) Types of study outcomes:
   a. the reported outcome can be assessed by Proctor’s implementation outcomes classified as acceptability, adoption, appropriateness, feasibility, penetration, and sustainability (3).
   b. Service outcome: efficiency, safety, effectiveness, equity, patient-centeredness, timeliness.
   c. Patient clinical outcomes will also be extracted: satisfaction, function, symptomatology.
9) Effect sizes: Eligible effect sizes (defined below, in the Analysis section) are those based on an eligible outcome from an eligible study design. The following effect sizes will be **excluded**:
   a. Effect sizes based on outcomes that are not compared between two study groups or are not compared over time in one study group (e.g., outcomes that are reported as the combined result of all study groups and thus no effect size can be calculated)
   b. Effect sizes based on a simulation study and not actually observed data
   c. Effect sizes based on measures of 100% at baseline and follow-up in the intervention group, as this indicates that HCP performance in the intervention group had no room for improvement and did not worsen over time. Similarly, for outcomes on HCP practices expressed as a percentage, we excluded effect sizes based on a baseline value of 95% or greater, as there was little room for improvement.
   d. Effect sizes based on outcome measures that were not taken at comparable times between study groups. For example, if the outcome for a control group was measured at –1 month, 3 months, and 9 months since the intervention began, and the outcome for an intervention group was measured at –1 month, 3 months, and 21 months since the intervention began, the effect size based on the 9-month and 21-month outcome measures would be ineligible.
   e. For ITS, we will exclude study outcomes for which the time series was highly unstable and thus could not be reliably modeled, and we will also exclude outlier outcome measures that probably did not represent the true trend in HCP performance (e.g., an unusually high baseline measure just before a strategy was implemented that was likely due to HCPs’ anticipation of the strategy).
10) Language: English.

**Supplementary Material 5: ERIC strategy category and sub-category**

| **#** | **ERIC Implementation Strategies** | **Definitions** |
| --- | --- | --- |
| **Use evaluative and iterative strategies** | |  |
| 4 | Assess for readiness and identify barriers and facilitators | Assess various aspects of an organization to determine its degree of readiness to implement, barriers that may impede implementation, and strengths that can be used in the implementation effort |
| 5 | Audit and provide feedback | Collect and summarize clinical performance data over a specified time period and give it to clinicians and administrators to monitor, evaluate, and modify provider behavior |
| 14 | Conduct cyclical small tests of change | Implement changes in a cyclical fashion using small tests of change before taking changes system-wide. Tests of change benefit from systematic measurement, and results of the tests of change are studied for insights on how to do better. This process continues serially over time, and refinement is added with each cycle |
| 18 | Conduct local need assessment | Collect and analyze data related to the need for the innovation |
| 23 | Develop a formal implementation blueprint | Develop a formal implementation blueprint that includes all goals and strategies. The blueprint should include the following: 1) aim/purpose of the implementation; 2) scope of the change (*e.g.*, what organizational units are affected); 3) timeframe and milestones; and 4) appropriate performance/progress measures. Use and update this plan to guide the implementation effort over time |
| 26 | Develop and implement tools for quality monitoring | Develop, test, and introduce into quality-monitoring systems the right input—the appropriate language, protocols, algorithms, standards, and measures (of processes, patient/consumer outcomes, and implementation outcomes) that are often specific to the innovation being implemented |
| 27 | Develop and organize quality monitoring systems | Develop and organize systems and procedures that monitor clinical processes and/or outcomes for the purpose of quality assurance and improvement |
| 46 | Obtain and use patients/consumers and family feedback | Develop strategies to increase patient/consumer and family feedback on the implementation effort |
| 56 | Purposefully reexamine the implementation | Monitor progress and adjust clinical practices and implementation strategies to continuously improve the quality of care |
| 61 | Stage implementation scale up | Phase implementation efforts by starting with small pilots or demonstration projects and gradually move to a system wide rollout |
| **Provide interactive assistance** | |  |
| 8 | Centralize technical assistance | Develop and use a centralized system to deliver technical assistance focused on implementation issues |
| 33 | Provide Implementation Facilitation | A process of interactive problem solving and support that occurs in a context of a recognized need for improvement and a supportive interpersonal relationship |
| 53 | Provide clinical supervision | Provide clinicians with ongoing supervision focusing on the innovation. Provide training for clinical supervisors who will supervise clinicians who provide the innovation |
| 54 | Provide local technical assistance | Develop and use a system to deliver technical assistance focused on implementation issues using local personnel |
| **Adapt and tailor to context** | |  |
| 51 | Promote adaptability | Identify the ways a clinical innovation can be tailored to meet local needs and clarify which elements of the innovation must be maintained to preserve fidelity |
| 63 | Tailor strategies | Tailor the implementation strategies to address barriers and leverage facilitators that were identified through earlier data collection |
| 67 | Use data experts | Involve, hire, and/or consult experts to inform management on the use of data generated by implementation efforts |
| 68 | Use data warehousing techniques | Integrate clinical records across facilities and organizations to facilitate implementation across systems |
| **Develop stakeholder interrelationships** | |  |
| 6 | Build a coalition | Recruit and cultivate relationships with partners in the implementation effort |
| 7 | Capture and share local knowledge | Capture local knowledge from implementation sites on how implementers and clinicians made something work in their setting and then share it with other sites |
| 17 | Conduct local consensus discussions | Include local providers and other stakeholders in discussions that address whether the chosen problem is important and whether the clinical innovation to address it is appropriate |
| 24 | Develop academic partnerships | Partner with a university or academic unit for the purposes of shared training and bringing research skills to an implementation project |
| 25 | Develop an implementation glossary | Develop and distribute a list of terms describing the innovation, implementation, and stakeholders in the organizational change |
| 35 | Identify and prepare champions | Identify and prepare individuals who dedicate themselves to supporting, marketing, and driving through an implementation, overcoming indifference or resistance that the intervention may provoke in an organization |
| 36 | Identify early adopters | Identify early adopters at the local site to learn from their experiences with the practice innovation |
| 38 | Inform local opinion leaders | Inform providers identified by colleagues as opinion leaders or “educationally influential” about the clinical innovation in the hopes that they will influence colleagues to adopt it |
| 40 | Involve executive boards | Involve existing governing structures (*e.g.*, boards of directors, medical staff boards of governance) in the implementation effort, including the review of data on implementation processes |
| 45 | Model and simulate change | Model or simulate the change that will be implemented prior to implementation |
| 47 | Obtain formal commitments | Obtain written commitments from key partners that state what they will do to implement the innovation |
| 48 | Organize clinician implementation team meetings | Develop and support teams of clinicians who are implementing the innovation and give them protected time to reflect on the implementation effort, share lessons learned, and support one another’s learning |
| 52 | Promote network weaving | Identify and build on existing high-quality working relationships and networks within and outside the organization, organizational units, teams, etc. to promote information sharing, collaborative problem-solving, and a shared vision/goal related to implementing the innovation |
| 57 | Recruit, designate, and train for leadership | Recruit, designate, and train leaders for the change effort |
| 64 | Use advisory boards and workgroups | Create and engage a formal group of multiple kinds of stakeholders to provide input and advice on implementation efforts and to elicit recommendations for improvements |
| 65 | Use an implementation advisor | Seek guidance from experts in implementation |
| 72 | Visit other sites | Visit sites where a similar implementation effort has been considered successful |
| **Train and educate stakeholders** | |  |
| 15 | Conduct educational meetings | Hold meetings targeted toward different stakeholder groups (*e.g.*, providers, administrators, other organizational stakeholders, and community, patient/consumer, and family stakeholders) to teach them about the clinical innovation |
| 16 | Conduct educational outreach visits | Have a trained person meet with providers in their practice settings to educate providers about the clinical innovation with the intent of changing the provider’s practice |
| 19 | Conduct ongoing training | Plan for and conduct training in the clinical innovation in an ongoing way |
| 20 | Create a learning collaborative | Facilitate the formation of groups of providers or provider organizations and foster a collaborative learning environment to improve implementation of the clinical innovation |
| 29 | Develop educational materials | Develop and format manuals, toolkits, and other supporting materials in ways that make it easier for stakeholders to learn about the innovation and for clinicians to learn how to deliver the clinical innovation |
| 31 | Distribute educational materials | Distribute educational materials (including guidelines, manuals, and toolkits) in person, by mail, and/or electronically |
| 43 | Make training dynamic | Vary the information delivery methods to cater to different learning styles and work contexts, and shape the training in the innovation to be interactive |
| 55 | Provide ongoing consultation | Provide ongoing consultation with one or more experts in the strategies used to support implementing the innovation |
| 60 | Shadow other experts | Provide ways for key individuals to directly observe experienced people engage with or use the targeted practice change/innovation |
| 71 | Use train-the-trainer strategies | Train designated clinicians or organizations to train others in the clinical innovation |
| 73 | Work with educational institutions | Encourage educational institutions to train clinicians in the innovation |
| **Support clinicians** | |  |
| 21 | Create new clinical teams | Change who serves on the clinical team, adding different disciplines and different skills to make it more likely that the clinical innovation is delivered (or is more successfully delivered) |
| 30 | Develop resource sharing agreements | Develop partnerships with organizations that have resources needed to implement the innovation |
| 32 | Facilitate relay of clinical data to providers | Provide as close to real-time data as possible about key measures of process/outcomes using integrated modes/channels of communication in a way that promotes use of the targeted innovation |
| 58 | Remind clinicians | Develop reminder systems designed to help clinicians to recall information and/or prompt them to use the clinical innovation |
| 59 | Revise professional roles | Shift and revise roles among professionals who provide care, and redesign job characteristics |
| **Engage consumers** | |  |
| 37 | Increase demand | Attempt to influence the market for the clinical innovation to increase competition intensity and to increase the maturity of the market for the clinical innovation |
| 39 | Intervene with patients/consumers to enhance uptake and adherence | Develop strategies with patients to encourage and problem solve around adherence |
| 41 | Involve patients/consumers and family members | Engage or include patients/consumers and families in the implementation effort |
| 50 | Prepare patients/consumers to be active participants | Prepare patients/consumers to be active in their care, to ask questions, and specifically to inquire about care guidelines, the evidence behind clinical decisions, or about available evidence-supported treatments |
| 69 | Use mass media | Use media to reach large numbers of people to spread the word about the clinical innovation |
| **Utilize financial strategies** | |  |
| 1 | Access new funding | Access new or existing money to facilitate the implementation |
| 2 | Alter incentive/allowance structures | Work to incentivize the adoption and implementation of the clinical innovation |
| 3 | Alter patient/consumer fees | Create fee structures where patients/consumers pay less for preferred treatments (the clinical innovation) and more for less-preferred treatments |
| 28 | Develop disincentives | Provide financial disincentives for failure to implement or use the clinical innovations |
| 34 | Fund and contract for the clinical innovation | Governments and other payers of services issue requests for proposals to deliver the innovation, use contracting processes to motivate providers to deliver the clinical innovation, and develop new funding formulas that make it more likely that providers will deliver the innovation |
| 42 | Make billing easier | Make it easier to bill for the clinical innovation |
| 49 | Place innovation on fee for service lists/formularies | Work to place the clinical innovation on lists of actions for which providers can be reimbursed (*e.g.*, a drug is placed on a formulary, a procedure is now reimbursable) |
| 66 | Use capitated payments | Pay providers or care systems a set amount per patient/consumer for delivering clinical care |
| 70 | Use other payment schemes | Introduce payment approaches (in a catch-all category) |
| **Change infrastructure** | |  |
| 9 | Change accreditation or membership requirements | Strive to alter accreditation standards so that they require or encourage use of the clinical innovation. Work to alter membership organization requirements so that those who want to affiliate with the organization are encouraged or required to use the clinical innovation |
| 10 | Change liability laws | Participate in liability reform efforts that make clinicians more willing to deliver the clinical innovation |
| 11 | Change physical structure and equipment | Evaluate current configurations and adapt, as needed, the physical structure and/or equipment (*e.g.*, changing the layout of a room, adding equipment) to best accommodate the targeted innovation |
| 12 | Change record systems | Change records systems to allow better assessment of implementation or clinical outcomes |
| 13 | Change service sites | Change the location of clinical service sites to increase access |
| 22 | Create or change credentialing and/or licensure standards | Create an organization that certifies clinicians in the innovation or encourage an existing organization to do so. Change governmental professional certification or licensure requirements to include delivering the innovation. Work to alter continuing education requirements to shape professional practice toward the innovation |
| 44 | Mandate change | Have leadership declare the priority of the innovation and their determination to have it implemented |
| 62 | Start a dissemination organization | Identify or start a separate organization that is responsible for disseminating the clinical innovation. It could be a for-profit or non-profit organization |

**Supplementary Material 6: Methodological details**

Methodologies for calculating the effect size

Effect size (ES) was used as the outcome measure in this study, which was defined as an absolute percentage-point difference. We used the calculation methods based on the study of Rowe et.al. A positive ES indicated improvement. We multiplied the ESs by –1 for study outcomes where a decrease indicated improvement. In this study, we included only percentage-based outcomes. The ES for the outcome A of a pre-post study with controls can be calculated as:

$Effect size=\left( Follow up-Baseline \right)_{i}-\left( Follow up-Baseline \right)_{c}$ (1)

*Follow up* can be the proportion with outcome A at follow-up.

*Baseline* can be the proportion with outcome A at baseline.

Meanwhile, many studies did not report baseline information, so the ES for a pre-post study with controls can be calculated as:

$Effect size=\left( \frac{{Follow up}_{i}-{Follow up}_{c}}{{Follow up}_{c}} \right)*100$ (2)

For meta-analysis based on risk difference, we also needed Standard Deviation (SD). Assuming statistical independence between study arms, variance of the effect size can be calculated as (*i* is the intervention group, *c* is the control group):

$Var \left( ES \right)=Var \left( {Follow up}_{i}-{Baseline}_{i} \right)+Var \left( {Follow up}_{c}-{Baseline}_{c} \right)$ (3)

Generally, $Var \left( Follow up-Baseline \right)=Var\left( Baseline \right)+Var\left( Follow up \right)-2*Cov(Follow up, Baseline)$ (4)

In formula (4),

$Var\left( Baseline \right)=Baseline*(1-Baseline)/n1$ (5)

$Var\left( Follow up \right)=Follow up*(1-Follow up)/n2$ (6)

$Cov\left( Follow up, Baseline \right)= correlation coefficient* \sqrt{Var\left( Baseline \right)*Var\left( Follow up \right)}$ (7)

In formula (5) and (6), n is the effective sample size at baseline or follow-up,

$n= number of people observed / design effect$ (8)

*design effect* in formula (8) can be calculated as:

$design effect= \{(intra- class correlation coefficient)* (\frac{number of people observed}{number of clusters observed}- 1)\} + 1$ (9)

Based on previous literature, intra-class correlation coefficient was assumed as 0.4 in formula (9); the correlation coefficient in formula (7) was assumed as 0.5.

The median effect size (MES) of all effect sizes in each study comparison was defined as the strategy effectiveness. The formula for the variance of a median effect size was also extracted based on the published article and was based on the theory of order:

$$Var\left( MES \right)=\pi*\frac{\sum variances of effect sizes within each comparison}{2*{(number of effect sizes within each comparison)}^{2}}$$

**Supplementary Material 7: List of excluded studies with reasons for exclusion**

Ineligible design

Hajebrahimi, S., Talebpour, A., Kabiri, N., Ahmadian, N., Soleimanzadeh, F., Behbahani, Z., & Salehi-Pourmehr, H. (2023). Promoting post discharge telephone follow-up of patients with transurethral resection for bladder cancer: a best practice implementation project. *JBI Evidence Implementation*, *21*(3), 251-258.

Hale, K., Capra, S., & Bauer, J. (2015). A Framework to Assist Health Professionals in Recommending High-Quality Apps for Supporting Chronic Disease Self-Management: Illustrative Assessment of Type 2 Diabetes Apps. *JMIR mHealth and uHealth*, *3*(3), e87. https://doi.org/10.2196/mhealth.4532

Koopman, R. J., Kochendorfer, K. M., Moore, J. L., Mehr, D. R., Wakefield, D. S., Yadamsuren, B., Coberly, J. S., Kruse, R. L., Wakefield, B. J., & Belden, J. L. (2011). A diabetes dashboard and physician efficiency and accuracy in accessing data needed for high-quality diabetes care. *Annals of family medicine*, *9*(5), 398–405. <https://doi.org/10.1370/afm.1286>

McCullagh, PJ., Mountain, GA., Black, N., Nugent, CD., Zheng, H., Davies, RJ., Galway, L., Hawley, MS., Mawson, SJ., Wright, P., Ecclesone, C., Nasr, N., & Parker, SJ. (2012). Knowledge transfer for technology based interventions: Collaboration, development and evaluation. *Technology and Disability*, *24*(3), 233-243. <https://doi.org/10.3233/TAD-2012-0349>

Besculides, M., Zaveri, H., Hanson, C., Farris, R., Gregory-Mercado, K., & Will, J. (2008). Best practices in implementing lifestyle interventions in the WISEWOMAN program: adaptable strategies for public health programs. *American journal of health promotion : AJHP*, *22*(5), 322–328. <https://doi.org/10.4278/ajhp.22.5.322>

Carter, J., Swack, N., Isselbacher, E., Donelan, K., & Thorndike, A. N. (2023). Feasibility and Acceptability of a Combined Digital Platform and Community Health Worker Intervention for Patients With Heart Failure: Single-Arm Pilot Study. *JMIR cardio*, *7*, e47818. <https://doi.org/10.2196/47818>

Cohn, W. F., Canan, C. E., Knight, S., Waldman, A. L., Dillingham, R., Ingersoll, K., Schexnayder, J., & Flickinger, T. E. (2021). An Implementation Strategy to Expand Mobile Health Use in HIV Care Settings: Rapid Evaluation Study Using the Consolidated Framework for Implementation Research. *JMIR mHealth and uHealth*, *9*(4), e19163. <https://doi.org/10.2196/19163>

Hazra-Ganju, A., Dlima, S. D., Menezes, S. R., Ganju, A., & Mer, A. (2023). An omni-channel, outcomes-focused approach to scale digital health interventions in resource-limited populations: a case study. *Frontiers in digital health*, *5*, 1007687. <https://doi.org/10.3389/fdgth.2023.1007687>

Jindal, D., Sharma, H., Gupta, Y. *et al.* Improving care for hypertension and diabetes in india by addition of clinical decision support system and task shifting in the national NCD program: I-TREC model of care. *BMC Health Serv Res* **22**, 688 (2022). https://doi.org/10.1186/s12913-022-08025-y

Jindal, D., Roy, A., Ajay, V. S., Yadav, S. K., Prabhakaran, D., & Tandon, N. (2019). Strategies for Stakeholder Engagement and Uptake of New Intervention: Experience From State-Wide Implementation of mHealth Technology for NCD Care in Tripura, India. *Global heart*, *14*(2), 165–172. <https://doi.org/10.1016/j.gheart.2019.06.002>

Ganz, D. A., Barnard, J. M., Smith, N. Z. Y., Miake-Lye, I. M., Delevan, D. M., Simon, A., Rose, D. E., Stockdale, S. E., Chang, E. T., Noël, P. H., Finley, E. P., Lee, M. L., Zulman, D. M., Cordasco, K. M., & Rubenstein, L. V. (2018). Development of a web-based toolkit to support improvement of care coordination in primary care. *Translational behavioral medicine*, *8*(3), 492–502. <https://doi.org/10.1093/tbm/ibx072>

De Raeve, P., Gomez, S., Hughes, P., Lyngholm, T., Sipilä, M., Kilanska, D., Hussey, P., Xyrichis, A., & ENS4Care project (2017). Enhancing the provision of health and social care in Europe through eHealth. *International nursing review*, *64*(1), 33–41. <https://doi.org/10.1111/inr.12266>

de Peralta, S. S., Ziaeian, B., Chang, D. S., Goldberg, S., Vetrivel, R., & Fang, Y. M. (2021). Leveraging telemedicine for management of veterans with heart failure during COVID-19. *Journal of the American Association of Nurse Practitioners*, *34*(1), 182–187. <https://doi.org/10.1097/JXX.0000000000000573>

Andersen, T., Bjørn, P., Kensing, F., & Moll, J. (2011). Designing for collaborative interpretation in telemonitoring: re-introducing patients as diagnostic agents. *International journal of medical informatics*, *80*(8), e112–e126. <https://doi.org/10.1016/j.ijmedinf.2010.09.010>

Cleveringa, F. G., Gorter, K. J., van den Donk, M., van Gijsel, J., & Rutten, G. E. (2013). Computerized decision support systems in primary care for type 2 diabetes patients only improve patients' outcomes when combined with feedback on performance and case management: a systematic review. *Diabetes technology & therapeutics*, *15*(2), 180–192. <https://doi.org/10.1089/dia.2012.0201>

Wise, M., Gustafson, D. H., Sorkness, C. A., Molfenter, T., Staresinic, A., Meis, T., Hawkins, R. P., Shanovich, K. K., & Walker, N. P. (2007). Internet telehealth for pediatric asthma case management: integrating computerized and case manager features for tailoring a Web-based asthma education program. *Health promotion practice*, *8*(3), 282–291. <https://doi.org/10.1177/1524839906289983>

Kedir K, Okop K, Ayele F M, et al. High rates of voluntary clinic attendance among community members screened with high cardiovascular disease risk scores in the rural and urban communities of Adama, Ethiopia[J]. BMC Public Health, 2025, 25(1): 3566.

Ostrem S, Stromberg A, Gjeilo K H, et al. The process of self-care maintenance in patients with heart failure following a nurse-assisted remote patient monitoring intervention: A qualitative longitudinal approach[J]. European Journal of Cardiovascular Nursing, 2025, 24(Supplement_1): zvaf122. 080.

No implementation strategies

Ehrhardt, N., Bouchonville, M., Peek, M. E., Thomas, C. C., Zou, T., Cuttriss, N., Desimone, M., Weinstock, R. S., Baer, L. G., & Gabbay, R. A. (2023). Telementoring With Project ECHO: A New Era in Diabetes-Related Continuing Education for Primary Care to Address Health Disparities. *Journal of diabetes science and technology*, *17*(4), 916–924. <https://doi.org/10.1177/19322968231155150>

Estrada, C. A., Safford, M. M., Salanitro, A. H., Houston, T. K., Curry, W., Williams, J. H., Ovalle, F., Kim, Y., Foster, P., & Allison, J. J. (2011). A web-based diabetes intervention for physician: a cluster-randomized effectiveness trial. *International journal for quality in health care : journal of the International Society for Quality in Health Care*, *23*(6), 682–689. <https://doi.org/10.1093/intqhc/mzr053>

Chambers, E. C., Wylie-Rosett, J., Blank, A. E., Ouziel, J., Hollingsworth, N., Riley, R. W., & Selwyn, P. A. (2015). Increasing Referrals to a YMCA-Based Diabetes Prevention Program: Effects of Electronic Referral System Modification and Provider Education in Federally Qualified Health Centers. *Preventing chronic disease*, *12*, E189. <https://doi.org/10.5888/pcd12.150294>

Cleveringa, F. G., Gorter, K. J., van den Donk, M., & Rutten, G. E. (2008). Combined task delegation, computerized decision support, and feedback improve cardiovascular risk for type 2 diabetic patients: a cluster randomized trial in primary care. *Diabetes care*, *31*(12), 2273–2275. <https://doi.org/10.2337/dc08-0312>

Vaughan, E. M., Naik, A. D., Amspoker, A. B., Johnston, C. A., Landrum, J. D., Balasubramanyam, A., Virani, S. S., Ballantyne, C. M., & Foreyt, J. P. (2021). Mentored implementation to initiate a diabetes program in an underserved community: a pilot study. *BMJ open diabetes research & care*, *9*(1), e002320. <https://doi.org/10.1136/bmjdrc-2021-002320>

Weber, V., Bloom, F., Pierdon, S., & Wood, C. (2008). Employing the electronic health record to improve diabetes care: a multifaceted intervention in an integrated delivery system. *Journal of general internal medicine*, *23*(4), 379–382. <https://doi.org/10.1007/s11606-007-0439-2>

Vaughan, E. M., Cardenas, V. J., Jr, Chan, W., Amspoker, A. B., Johnston, C. A., Virani, S. S., Ballantyne, C. M., & Naik, A. D. (2024). Implementation and Evaluation of a mHealth-Based Community Health Worker Feedback Loop for Hispanics with and at Risk for Diabetes. *Journal of general internal medicine*, *39*(2), 229–238. <https://doi.org/10.1007/s11606-023-08434-7>

Carron, T., Bridevaux, P. O., Lörvall, K., Parmentier, R., Moix, J. B., Beytrison, V., Pernet, R., Rey, C., Roberfroid, P. Y., Chhajed, P. N., Dieterle, T., Joos Zellweger, L., Kohler, M., Maier, S., Miedinger, D., Thurnheer, R., Urwyler, P., Tschopp, J. M., Zuercher, E., Leuppi, J. D., … Peytremann-Bridevaux, I. (2017). Feasibility, acceptability and effectiveness of integrated care for COPD patients: a mixed methods evaluation of a pilot community-based programme. *Swiss medical weekly*, *147*, w14567. <https://doi.org/10.4414/smw.2017.14567>

Sim, L. L., Ban, K. H., Tan, T. W., Sethi, S. K., & Loh, T. P. (2017). Development of a clinical decision support system for diabetes care: A pilot study. *PloS one*, *12*(2), e0173021. <https://doi.org/10.1371/journal.pone.0173021>

Sequist, T. D., Gandhi, T. K., Karson, A. S., Fiskio, J. M., Bugbee, D., Sperling, M., Cook, E. F., Orav, E. J., Fairchild, D. G., & Bates, D. W. (2005). A randomized trial of electronic clinical reminders to improve quality of care for diabetes and coronary artery disease. *Journal of the American Medical Informatics Association : JAMIA*, *12*(4), 431–437. <https://doi.org/10.1197/jamia.M1788>

Samuel-Hodge, C. D., Gizlice, Z., Allgood, S. D., Bunton, A. J., Erskine, A., Leeman, J., & Cykert, S. (2020). Strengthening community-clinical linkages to reduce cardiovascular disease risk in rural NC: feasibility phase of the CHANGE study. *BMC public health*, *20*(1), 264. <https://doi.org/10.1186/s12889-020-8223-x>

Leng L, Arévalo Avalos M R, Aguilera A, et al. The Impact of an Adaptive mHealth Intervention on Improving Patient-Provider Health Care Communication: Secondary Analysis of the DIAMANTE Trial[J]. JMIR mHealth and uHealth, 2025, 13: e64296.

Lin J, Huber B, Amir O, et al. Novel Web-Based Technology to Promote Goal-Setting in Complex Chronic Illness: Randomized Controlled Trial[J]. JMIR Human Factors, 2026, 13: e70402.

Caballero Mateos I, Morales Portillo C, Lainez López M, et al. Efficacy of a digital educational intervention for patients with type 2 diabetes mellitus: multicenter, randomized, prospective, 6-month follow-up study[J]. Journal of Medical Internet Research, 2025, 27: e60758.

Mira-Martínez S, Malih N, Angullo-Martínez E, et al. A Multifaceted Digital Intervention for the Prevention of Type 2 Diabetes Mellitus in Primary Care (PREDIABETEXT): Cluster Randomized Trial[J]. Journal of Medical Internet Research, 2025, 27: e70981.

Mohammadi-Avizi D, Firouzkouhi M, Abdollahimohammad A, et al. Comparison of the effect of patient-centred and family-centred education through smartphones on the quality of life of patients with type 2 diabetes: A quasi-experimental study[J]. Healthcare in Low-Resource Settings, 2024, 12(2).

Nguyen P V N, Thach P T, Tran H K, et al. Effectiveness of a digital diabetes self-management education and support program in Vietnamese adults with type 2 diabetes[J]. Scientific Reports, 2025, 15(1): 41369.

Obilor H N, Veryha O, Weisz T, et al. The feasibility of a social media-based foot self-management education and support program for adults with diabetes: A partially randomized preference trial[J]. PEC innovation, 2024, 5: 100307.

Pastrana E, Hilario E M. The Effect of Inhaler Device Handling Education Using Video Tutorial in Comparison to Physician-Led Teaching in COPD and Asthmatic Patients: A Randomized Controlled Trial[C]//RESPIROLOGY. 111 RIVER ST, HOBOKEN 07030-5774, NJ USA: WILEY, 2025, 30.

Rotimi-Oyedepo V O, Samson-Akpan P E, Olofin-Samuel M A. Effect of intervention on preventive practices of hypertension and diabetes among teachers[J]. Journal of Public Health in Africa, 2026, 17(1): 1418.

Rustambekova A R, Moldomamatova A I, Askarbekova K A, et al. Remote monitoring of patients with NYHA functional class II-III chronic heart failure using the M-Cardio mobile application in residents of Kyrgyzstan[J]. Kardiologiia, 2025, 65(6): 44-53.

Schultz A N Ø, Christensen R, Bollig G, et al. Effectiveness of video consultations in type 1 diabetes patients treated with insulin pumps in the outpatient clinic: a randomised controlled trial[J]. Diabetologia, 2026, 69(2): 321-329.

Shah M K, Wyatt L C, Gibbs-Tewary C, et al. A Culturally Adapted, Telehealth, Community Health Worker Intervention on Blood Pressure Control among South Asian Immigrants with Type II Diabetes: Results from the DREAM Atlanta Intervention: Shah et al[J]. Journal of General Internal Medicine, 2024, 39(4): 529-539.

Shi Y, Sevick M A, Tang H, et al. Culturally Tailored Social Media Intervention Improves Health Outcomes in Chinese Americans with Type 2 Diabetes: Preliminary Evidence from a Pilot RCT: Shi et al[J]. Journal of General Internal Medicine, 2025, 40(10): 2319-2329.

Sten-Gahmberg S, Pedersen K, Harsheim I G, et al. Pragmatic randomized controlled trial comparing a complex telemedicine-based intervention with usual care in patients with chronic conditions: S. Sten-Gahmberg et al[J]. The European Journal of Health Economics, 2024, 25(7): 1275-1289.

Sterling M R, Espinosa C G, Vergez S, et al. Home health aides caring for adults with heart failure: a pilot randomized clinical trial[J]. JAMA Network Open, 2025, 8(11): e2548121.

Tan N C, Tyagi S, Lee C S, et al. Effectiveness of an algorithm-driven home telemonitoring system on the metabolic control and self-care behaviour of Asian adults with type-2 diabetes mellitus: A randomised controlled trial[J]. Journal of Telemedicine and Telecare, 2025, 31(4): 523-533.

Xu W, Huang X, Lin Q, et al. Application of Alfalfa App in the management of oral anticoagulation in patients with atrial fibrillation: a multicenter randomized controlled trial[J]. BMC medical informatics and decision making, 2024, 24(1): 294.

Yamashita S, Katsumata Y, Kohsaka S, et al. Electronic patient-reported outcome system implementation in outpatient cardiovascular care: a randomized clinical trial[J]. JAMA network open, 2025, 8(1): e2454084.

Yu J, Lee J, Yang Y, et al. Clinical Impact of Personalized Physician’s Education and Remote Feedback Via a Digital Platform on Glycemic Control: Pilot Randomized Controlled Trial[J]. JMIR mHealth and uHealth, 2025, 13(1): e67151.

Yun S, Comín-Colet J, Calero-Molina E, et al. Evaluation of mobile health technology combining telemonitoring and teleintervention versus usual care in vulnerable-phase heart failure management (HERMeS): a multicentre, randomised controlled trial[J]. The Lancet Digital Health, 2025, 7(5).

Zhang X, Wang S, Zhou X, et al. A village doctor-led mobile health intervention for cardiovascular risk reduction in rural China: cluster randomised controlled trial[J]. bmj, 2025, 389.

Not related to providers’ behaviors

Bosworth, H. B., Powers, B. J., Olsen, M. K., McCant, F., Grubber, J., Smith, V., Gentry, P. W., Rose, C., Van Houtven, C., Wang, V., Goldstein, M. K., & Oddone, E. Z. (2011). Home blood pressure management and improved blood pressure control: results from a randomized controlled trial. *Archives of internal medicine*, *171*(13), 1173–1180. <https://doi.org/10.1001/archinternmed.2011.276>

Lee, Y. B., Kim, G., Jun, J. E., Park, H., Lee, W. J., Hwang, Y. C., & Kim, J. H. (2023). An Integrated Digital Health Care Platform for Diabetes Management With AI-Based Dietary Management: 48-Week Results From a Randomized Controlled Trial. *Diabetes care*, *46*(5), 959–966. <https://doi.org/10.2337/dc22-1929>

Piette, J. D., Marinec, N., Janda, K., Morgan, E., Schantz, K., Yujra, A. C., Pinto, B., Soto, J. M., Janevic, M., & Aikens, J. E. (2016). Structured Caregiver Feedback Enhances Engagement and Impact of Mobile Health Support: A Randomized Trial in a Lower-Middle-Income Country. *Telemedicine journal and e-health : the official journal of the American Telemedicine Association*, *22*(4), 261–268. <https://doi.org/10.1089/tmj.2015.0099>

Persell, S. D., Karmali, K. N., Lazar, D., Friesema, E. M., Lee, J. Y., Rademaker, A., Kaiser, D., Eder, M., French, D. D., Brown, T., & Wolf, M. S. (2018). Effect of Electronic Health Record-Based Medication Support and Nurse-Led Medication Therapy Management on Hypertension and Medication Self-management: A Randomized Clinical Trial. *JAMA internal medicine*, *178*(8), 1069–1077. <https://doi.org/10.1001/jamainternmed.2018.2372>

Williams, S. L., To, Q., & Vandelanotte, C. (2022). What is the effectiveness of a personalised video story after an online diabetes risk assessment? A Randomised Controlled Trial. *PloS one*, *17*(3), e0264749. <https://doi.org/10.1371/journal.pone.0264749>

Poulter S E, Meloncelli N, Mack M. Use of a smartphone-based, interactive blood glucose management system in women with gestational diabetes mellitus: a pilot study[J]. Diabetes Research and Clinical Practice, 2022, 185: 109224.

Devaraju C J, Sunil Kumar S, Sadananda K S, et al. Impact of Digital Technology Intervention on Medication Compliance, Modification of Risk Factors, and Clinical Outcome in Patients With Acute Coronary Syndrome: A Follow-Up Study[J]. International Journal of Cardiovascular Sciences, 2025, 38: e20240127.

Eynan R, Petrella R, Forchuk C, et al. Randomised pilot study comparing a coach to SMARTPhone reminders to aid the management of heart failure (HF) patients: humans or machines[J]. BMJ Open Quality, 2024, 13(3).

FarzanehRad A, Allahbakhshian A, Gholizadeh L, et al. Randomized comparison of the effects of tailored text messaging versus pillbox organizers on medication adherence of heart failure patients[J]. BMC Cardiovascular Disorders, 2024, 24(1): 244.

Favaretti C, Subramonia Pillai V, Murthy S, et al. Effectiveness of WhatsApp based debunking reminders on follow-up visit attendance for individuals with hypertension: a randomized controlled trial in India[J]. BMC Public Health, 2024, 24(1): 2441.

Giovanazzi A, Gios L, Gentilini M A, et al. Impact of digital health on Type 2 diabetes management: a randomised controlled trial of the ‘TreC Diabete’platform (TELEMECHRON Study)[J]. Frontiers in Clinical Diabetes and Healthcare, 2025, 6: 1589548.

Glavas C, Scott D, Sood S, et al. Exploring the feasibility of digital voice assistants for delivery of a home-based exercise intervention in older adults with obesity and type 2 diabetes mellitus: randomized controlled trial[J]. JMIR aging, 2024, 7(1): e53064.

Ha S K, Gilbert J B, Le E, et al. Impact of teleretinal screening program on diabetic retinopathy screening compliance rates in community health centers: a quasi-experimental study[J]. BMC Health Services Research, 2025, 25(1): 318.

Hickey M D, Owaraganise A, Ogachi S, et al. Community health worker–facilitated telehealth for moderate–severe hypertension care in Kenya and Uganda: A randomized controlled trial[J]. PLoS Medicine, 2025, 22(6): e1004632.

Holloway E E, Jenkins L, Agius P A, et al. Effectiveness of low intensity mental health support via a telehealth enabled network (LISTEN) for adults with diabetes distress: A parallel group, pragmatic randomized controlled trial[J]. Diabetes Care, 2025, 48(6): 955-965.

Vaughan E M, Yu X, Cardenas Jr V J, et al. From trials to practice: implementing a clinical intervention in community settings[J]. Journal of Primary Care & Community Health, 2025, 16: 21501319251339190.

Ineligible comparison groups

Fricton, J., Rindal, D. B., Rush, W., Flottemesch, T., Vazquez, G., Thoele, M. J., Durand, E., Enstad, C., & Rhodus, N. (2011). The effect of electronic health records on the use of clinical care guidelines for patients with medically complex conditions. *Journal of the American Dental Association (1939)*, *142*(10), 1133–1142. <https://doi.org/10.14219/jada.archive.2011.0082>

Sánchez, A., Silvestre, C., Campo, N., Grandes, G., & PreDE research group (2016). Type-2 diabetes primary prevention program implemented in routine primary care: a process evaluation study. *Trials*, *17*(1), 254. <https://doi.org/10.1186/s13063-016-1379-0>

Sciamanna, C. N., Gifford, D. R., & Smith, R. J. (2004). Design and acceptability of patient-oriented computerized diabetes care reminders for use at the point of care. *Medical informatics and the Internet in medicine*, *29*(2), 157–168. <https://doi.org/10.1080/14639230412331280413>

Ineligible disease

Thomas, R. E., Croal, B. L., Ramsay, C., Eccles, M., & Grimshaw, J. (2006). Effect of enhanced feedback and brief educational reminder messages on laboratory test requesting in primary care: a cluster randomised trial. *Lancet (London, England)*, *367*(9527), 1990–1996. <https://doi.org/10.1016/S0140-6736(06)68888-0>

Simon, G. E., VonKorff, M., Rutter, C., & Wagner, E. (2000). Randomised trial of monitoring, feedback, and management of care by telephone to improve treatment of depression in primary care. *BMJ (Clinical research ed.)*, *320*(7234), 550–554. <https://doi.org/10.1136/bmj.320.7234.550>

Fortuna, R. J., Zhang, F., Ross-Degnan, D., Campion, F. X., Finkelstein, J. A., Kotch, J. B., Feldstein, A. C., Smith, D. H., & Simon, S. R. (2009). Reducing the prescribing of heavily marketed medications: a randomized controlled trial. *Journal of general internal medicine*, *24*(8), 897–903. <https://doi.org/10.1007/s11606-009-1013-x>

Beidas, R. S., Linn, K. A., Boggs, J. M., Marcus, S. C., Hoskins, K., Jager-Hyman, S., Johnson, C., Maye, M., Quintana, L., Wolk, C. B., Wright, L., Pappas, C., Beck, A., Bedjeti, K., Buttenheim, A. M., Daley, M. F., Elias, M., Lyons, J., Martin, M. L., McArdle, B., … Ahmedani, B. K. (2024). Implementation of a Secure Firearm Storage Program in Pediatric Primary Care: A Cluster Randomized Trial. *JAMA pediatrics*, *178*(11), 1104–1113. <https://doi.org/10.1001/jamapediatrics.2024.3274>

Ineligible study outcomes

Lowres, N., Duckworth, A., Redfern, J., Thiagalingam, A., & Chow, C. K. (2020). Use of a Machine Learning Program to Correctly Triage Incoming Text Messaging Replies From a Cardiovascular Text-Based Secondary Prevention Program: Feasibility Study. *JMIR mHealth and uHealth*, *8*(6), e19200. <https://doi.org/10.2196/19200>

Mollon, B., Holbrook, A. M., Keshavjee, K., Troyan, S., Gaebel, K., Thabane, L., & Perera, G. (2008). Automated telephone reminder messages can assist electronic diabetes care. *Journal of telemedicine and telecare*, *14*(1), 32–36. <https://doi.org/10.1258/jtt.2007.070702>

Thompson, J. S., Fitzgerald, M. D., Allen, L. A., McIlvennan, C. K., Glasgow, R. E., Wynia, M., Morris, M. A., Beilenson, J., Gherst, C., & Matlock, D. D. (2021). Shared Decision-Making for Left Ventricular Assist Devices: Rationale and Design of a Nationwide Dissemination and Implementation Project. *Circulation. Cardiovascular quality and outcomes*, *14*(2), e007256. <https://doi.org/10.1161/CIRCOUTCOMES.120.007256>

No digital health technologies

Sanders, K. M., & Satyvavolu, A. (2002). Improving blood pressure control in diabetes: limitations of a clinical reminder in influencing physician behavior. *The Journal of continuing education in the health professions*, *22*(1), 23–32. <https://doi.org/10.1002/chp.1340220104>

Weiner, S., Schwartz, A., Altman, L., Ball, S., Bartle, B., Binns-Calvey, A., Chan, C., Falck-Ytter, C., Frenchman, M., Gee, B., Jackson, J. L., Jordan, N., Kass, B., Kelly, B., Safdar, N., Scholcoff, C., Sharma, G., Weaver, F., & Wopat, M. (2020). Evaluation of a Patient-Collected Audio Audit and Feedback Quality Improvement Program on Clinician Attention to Patient Life Context and Health Care Costs in the Veterans Affairs Health Care System. *JAMA network open*, *3*(7), e209644. <https://doi.org/10.1001/jamanetworkopen.2020.9644>

Kwan B M, Dickinson L M, Dailey-Vail J, et al. Comparative Effectiveness of Patient-Driven versus Standardized Diabetes Shared Medical Appointments: A Pragmatic Cluster Randomized Trial: Kwan et al[J]. Journal of General Internal Medicine, 2024, 39(15): 2970-2979.

Abd Alrahman M S, Jasim A L, Alhilal M A. Enhancing Adherence and Satisfaction with Insulin Therapy in Type 1 Diabetes Mellitus Patients Through Pharmacist-Led Interventions: Interventional study[J]. Bahrain Medical Bulletin, 2025, 47(2).

Amin S M, Ghallab E, Dreidi M, et al. Impact of the Diabetes Conversation MapTM Program on Knowledge Retention, Self‐Management, and Self‐Efficacy Among Diabetic Patients: A Randomized Controlled Trial[J]. Worldviews on Evidence‐Based Nursing, 2025, 22(4): e70055.

Andriessen C, Blom M T, van Hoek B A C E, et al. Feasibility of implementing an intervention in general practice for deprescribing of glucose-lowering medication in overtreated elderly[J]. Family practice, 2025, 42(5): cmaf064.

de Goes Marques C R, Santos E S, da Silva L S, et al. Effectiveness of an Educational Intervention With Home Visits and Telephone Follow‐Up on Knowledge and Quality of Life in Patients With Heart Failure: A Randomized Clinical Trial[J]. Nursing & Health Sciences, 2026, 28(1): e70309.

Zou C, Deng L, Luo J, et al. The impact of communication training on the clinical care of hypertension in general practice: a cluster randomized controlled trial in China[J]. BMC Primary Care, 2024, 25(1): 98.

Study protocol

Zullig, L. L., Shahsahebi, M., Neely, B., Hyslop, T., Avecilla, R. A. V., Griffin, B. M., Clayton-Stiglbauer, K., Coles, T., Owen, L., Reeve, B. B., Shah, K., Shelby, R. A., Sutton, L., Dinan, M. A., Zafar, S. Y., Shah, N. P., Dent, S., & Oeffinger, K. C. (2021). Low-touch, team-based care for co-morbidity management in cancer patients: the ONE TEAM randomized controlled trial. *BMC family practice*, *22*(1), 234. <https://doi.org/10.1186/s12875-021-01569-8>

Byun J Y, Wells R, Bechthold A C, et al. Project EPIC (Empowering People to Independence in COPD): Study protocol for a hybrid effectiveness-implementation pilot randomized controlled trial of telephonic, geriatrics-palliative care nurse-coaching in older adults with COPD and their family caregivers[J]. Contemporary clinical trials, 2024, 140: 107487.

Ghavami M, Sadeghian S, Ahmadi A, et al. The effectiveness of green heart application to manage modifiable risk factors of coronary artery disease in Tehran Heart Center: Study protocol for a randomized controlled trial[J]. Heliyon, 2024, 10(7).

Hobden B, Fakes K, Halcomb E J, et al. Wearables Integrated Technology to support healthy behaviours in general practice patients with type 2 diabetes (WEAR-IT): study protocol for a cluster randomised controlled trial[J]. BMJ open, 2025, 15(10): e105399.

Homs A F, Lachaux R, Vallayer V, et al. Effects of a remote therapeutic education programme involving peers and health professionals on physical activity in patients with coronary heart disease undergoing phase 3 cardiac rehabilitation: protocol for a single-centre randomised controlled trial[J]. BMJ open, 2025, 15(6): e095196.

Johnson E, Ismond K, Hyde A, et al. eMPower: An online Mind-body wellness Program for adults living with chronic health conditions: A three-armed randomized controlled trial protocol[J]. Contemporary Clinical Trials, 2024, 145: 107642.

Preprint

Barber K, Deaton C, McCann G P, et al. Think-HF: Development, feasibility, and usability of an electronic health record integrated tool for identifying missed diagnoses of heart failure in primary care[J]. medRxiv, 2026: 2026.01. 23.26344713.

Firuz U, Dadaxon A, Zafar N, et al. Efficacy of digital health applications in improving treatment adherence among patients with resistant hypertension: a randomized controlled trial[J]. Revista Latinoamericana de Hipertensión, 2025, 20(7).

Conference

Miller H, Askew S, Berger M, et al. Digital Health Intervention Yields Modest Improvements in Diet and Blood Pressure Among Adults with Hypertension[J]. Circulation, 2024, 150(Suppl_1): A4144010-A4144010.

Pikel K, Burger K, Menon S, et al. Population Health Approach to Blood Pressure Control Using Advanced Practice Provider-Managed Remote Patient Monitoring[J]. The Journal for Healthcare Quality (JHQ), 2022: 10.1097.

Raffoul N, Brims K, Knight J, et al. Embedding a text message recall system to increase the uptake of cardiovascular screening in Australian general practice. Results of the Text to Detect randomised controlled trial[J]. European Heart Journal, 2024, 45(Supplement_1): ehae666. 3597.

Wismer G, Batio S, Kwasny M, et al. REGIMEN EDUCATION AND MESSAGING IN DIABETES, THE REMIND TRIAL[C]//JOURNAL OF GENERAL INTERNAL MEDICINE. ONE NEW YORK PLAZA, SUITE 4600, NEW YORK, NY, UNITED STATES: SPRINGER, 2024, 39: S656-S657.

Yu D, Lau J C C, Keung G, et al. Effects of a dyadic empowerment-based heart failure management program (De-HF) on patient-reported and clinical outcomes: a double-blind randomized controlled trial[J]. European Heart Journal, 2025, 46(Supplement_1): ehaf784. 1310.

**Supplementary Material 8: Characteristics of the included studies (N=32)**

| **Study design** | Number of studies (%) |
| --- | --- |
| Cluster randomized trial | 19 (59.4) |
| Randomized controlled trial | 9 (28.1) |
| Interrupted time series with randomized controls | 4 (12.5) |
| **WHO region where study was conducted** |  |
| America | 25 (78.1) |
| Europe | 4 (12.5) |
| Western Pacific | 3 (9.4) |
| **Year of publication** |  |
| 2020 - 2025 | 7 (21.9) |
| 2010 - 2019 | 10 (31.3) |
| 2000–2009 | 15 (46.9) |
| **Data collection methods (multiple responses allowed per study)** |  |
| Record or chart review | 24 (75.0) |
| Interview with patients or their caregivers | 4 (12.5) |
| Questionnaire for Health Care Providers | 4 (12.5) |
| **Urban vs. rural study setting** |  |
| Urban | 7 (21.9) |
| Rural | 4 (12.5) |
| Urban+Rural | 1 (3.1) |
| Not reported | 20 (62.5) |
| **Places where services were delivered (multiple responses allowed)** | |
| Outpatient health facility | 20 (62.5) |
| Hospital inpatient wards | 7 (21.9) |
| Mixed | 3 (9.4) |
| Hospital outpatient department | 2 (6.3) |
| **Who owns or operates the place where services were delivered** |  |
| Public or government | 13 (40.6) |
| Private, not for profit | 8 (25.0) |
| Unclear or not reported | 7 (21.9) |
| Private, profit status unknown or not reported | 2 (6.3) |
| mixed | 2 (6.3) |
| **Type of health care providers (multiple responses allowed per study)** | |
| Physician | 20 (62.5) |
| Multiple Health Care Providers | 10 (31.3) |
| Nurse | 1 (3.1) |
| Clinical officer | 1 (3.1) |
| **Chronic condition (multiple responses allowed per study)** |  |
| Diabetes | 10 (31.3) |
| Cardiovascular disease | 7 (21.9) |
| Multiple chronic conditions | 7 (21.9) |
| Chronic Kidney Disease | 2 (6.3) |
| Disability | 1 (3.1) |
| Frailty | 1 (3.1) |
| Dyslipidemia | 1 (3.1) |
| Hypertension | 1 (3.1) |
| Osteoporosis | 1 (3.1) |
| Multiple sclerosis | 1 (3.1) |
| **DHTs category** |  |
| Clinical reminder | 10 (31.3) |
| Computerized Decision Support System | 8 (25.0) |
| e-Health | 6 (18.8) |
| Telemedicine | 5 (15.6) |
| EMR | 3 (9.4) |
| **HCP Behavior (multiple responses allowed per study)** |  |
| Treatment | 23 (71.9) |
| Patient assessment | 8 (25.0) |
| Referral | 3 (9.4) |
| Documentation | 3 (9.4) |
| Diagnosis | 2 (6.3) |
| Counseling | 2 (6.3) |
| Vaccination | 2 (6.3) |

**Supplementary Material 9: Risk of bias**

Cluster RCT

RCT

NRCS

**Supplementary Material 10: GRADE scoring process and results**

GRADE process for standardized meta-analysis

Since most included studies were randomized controlled trials (RCTs) or cluster randomized trials (CRTs), all comparisons initially started with a high-quality rating. Then, we considered downgrading the evidence based on five factors:

| **Factor** | **Rules** |
| --- | --- |
| Risk of Bias | Downgrade if >50% of included studies have a high risk of bias |
| Inconsistency | Downgrade if results contained high heterogeneity (I² > 50%) without explanation |
| Indirectness | Downgrade if the study population, intervention, or outcome differs from the clinical question |
| Imprecision | Downgrade if CIs include both benefit and harm |
| Publication Bias | Downgrade if strong suspicion of publication bias. (e.g., asymmetric funnel plot or significant Egger’s test) |

Additionally, we considered upgrading the evidence based on two factors:

| **Factor** | **How It Affects GRADE Rating** |
| --- | --- |
| Large Effect Size | Upgrade if RD > 10% (0.1) without confounding |
| Dose-Response | Upgrade if increasing exposure is linked to a stronger effect |

GRADE process for network meta-analysis (NMA)

A systematic four-step approach was employed in this study to assess the quality of evidence. First, the effect sizes and confidence intervals for direct evidence, indirect evidence, and network meta-analysis (NMA) evidence were reported separately. Since most included studies were randomized controlled trials (RCTs) or cluster randomized trials (CRTs), all comparisons initially started with a high-quality rating. Second, the quality of direct evidence for each comparison was assessed without considering imprecision. If the direct evidence was graded as high and its contribution to the NMA results was greater than or equal to that of the indirect evidence, no further grading of indirect evidence was required, and the NMA evidence quality was directly derived from the direct evidence quality. Otherwise, an additional assessment of indirect evidence quality was necessary. Third, the quality of indirect evidence was determined while also considering intransitivity—potential differences across studies that could affect the validity of indirect comparisons. Finally, the overall quality of NMA evidence was determined based on the ratings of direct and/or indirect evidence, while also accounting for inconsistency and imprecision. The final quality ratings were then presented accordingly. This structured approach ensured a transparent and rigorous evaluation of the evidence in the network meta-analysis.

***Serious risk of bias:*** In this study, the comparisons were downgraded if >50% of included studies have a high risk of bias

***Serious Indirectness:*** The comparisons with no direct evidence were downgraded due to the existence of Indirectness.

***Intransitivity:*** When dealing with indirect evidence quality grading, it is necessary to pay attention to whether there are significant differences between different groups in terms of population baseline characteristics, common controls, and outcome measures. Therefore, most comparisons were downgraded in the intransitivity of evidence due to the differences in population (e.g. age and sex) and outcome (outcome subtypes in this study).

***Imprecision:*** Judgments of imprecision were made using a minimally contextualized approach, with a null effect as the threshold of importance. Imprecision was judged after the judgement of evidence contribution.

***Heterogeneity:*** Judged according to the pairwise meta-analysis from the network meta-analysis.

***Direct evidence and indirect evidence contribution:*** The GRADE working group recommends that the contribution of direct evidence and indirect evidence to NMA results be judged by the width of the confidence interval, and evidence with a narrower confidence interval contributes more to the NMA.

***Incoherence:*** Downgrade if there is significant inconsistency between direct and indirect comparative evidence.

GRADE results for standardized meta-analysis

| **Name** | **Number of studies** | **High risk studies** | **Inconsistency (heterogeneity)** | **Indirectness** | **Imprecision** | **Publication Bias** | **Evidence** | **Evidence level** |
| --- | --- | --- | --- | --- | --- | --- | --- | --- |
| ***Primary analysis, outcome type = Implementation, pooled analysis*** | | | | | | | | |
| Implementation strategies vs no active implementation strategy | 23 | 16† | 81.3%# | -- |  | § | 0.02 [0.00; 0.03] | ⨁⨁◯◯ |
| ***Primary analysis, outcome type = Implementation, intervention specific*** | | | | | | | | |
| Train & educate stakeholders vs no active implementation strategy | 4 | 4† | 79.3%* | -- |  | -- | 0.03 [0.00; 0.06] | ⨁⨁◯◯ |
| Multifaceted implementation strategy vs no active implementation strategy | 3 | 3† | 0% | -- | ⇟ | -- | 0.02 [-0.02; 0.05] | ⨁⨁◯◯ |
| Support clinicians vs no active implementation strategy | 6 | 4† | 80.7%* | -- | ⇟ | -- | 0.05 [-0.01; 0.12] | ⨁◯◯◯ |
| Change infrastructure vs No active implementation strategy | 2 | 2† | 20.2% | -- | ⇟ | -- | 0.00 [-0.02; 0.03] | ⨁⨁◯◯ |
| Develop stakeholder interrelationships vs no active implementation strategy | 1 | 0 | -- | -- | ⇟ | -- | 0.06 [-0.07; 0.20] | ⨁⨁⨁◯ |
| Engage consumers vs no active implementation strategy | 6 | 3 | 29.6%* | -- | ⇟ | -- | -0.01 [-0.08; 0.06] | ⨁⨁⨁◯ |
| Use evaluative & iterative strategies vs no active implementation strategy vs no active implementation strategy | 1 | 0 | -- | -- | ⇟ | -- | -0.01 [-0.03; 0.01] | ⨁⨁⨁◯ |
| ***Secondary analysis, outcome type = Patient*** | | | | | | | | |
| Implementation strategies vs no active implementation strategy | 3 | 1 | 32% | -- | ⇟ | -- | -0.01 [-0.03; 0.02] | ⨁⨁⨁◯ |
| ***Secondary analysis, outcome type = Service*** | | | | | | | | |
| Implementation strategies vs no active implementation strategy | 2 | 1 | 0% | -- | ⇟ | -- | -0.03 [-0.09; 0.04] | ⨁⨁⨁◯ |
| ***Subgroup analysis, outcome type = Implementation, by Healthcare provider (HCP)’s behavior*** | | | | | | | | |
| HCP behavior = counselling | 2 | 2† | 31.8% | -- | ⇟ | -- | 0.03 [-0.03; 0.10] | ⨁⨁◯◯ |
| HCP behavior = patient assessment | 8 | 5† | 72.9* | -- | ⇟ | -- | 0.01 [-0.06; 0.09] | ⨁◯◯◯ |
| HCP behavior = treatment | 15 | 10† | 55.8* | -- | ⇟ | -- | 0.00 [-0.00; 0.01] | ⨁◯◯◯ |
| HCP behavior = vaccination | 2 | 1 | 17% | -- | ⇟ | -- | 0.01 [-0.04; 0.06] | ⨁⨁⨁◯ |
| HCP behavior = documentation | 3 | 1 | 0% | -- |  | -- | 0.05 [0.04; 0.06] | ⨁⨁⨁⨁ |
| HCP behavior = diagnosis | 2 | 2† | 0% | -- |  | -- | 0.05 [0.04; 0.06] | ⨁⨁⨁◯ |
| HCP behavior = referral | 2 | 1 | 54.2%* | -- | ⇟ | -- | 0.03 [-0.06; 0.12] | ⨁⨁◯◯ |
| ***Subgroup analysis, outcome type = Implementation, by Number of components*** | | | | | | | | |
| Number of components = more than 1 component | 9 | 8† | 54.8%* | -- |  | -- | 0.03 [0.01; 0.05] | ⨁⨁◯◯ |
| Number of components = one component | 14 | 8† | 64.8%* | -- | ⇟ | -- | 0.00 [-0.00; 0.01] | ⨁◯◯◯ |
| ***Sensitivity analysis 1, outcome type = Implementation, component-inclusive sensitivity analysis*** | | | | | | | | |
| Develop stakeholder interrelationships vs no active implementation strategy | 3 | 1 | 0% | -- | ⇟ | -- | 0.02 [-0.03; 0.07] | ⨁⨁⨁◯ |
| Support clinicians vs no active implementation strategy | 7 | 5† | 77.5%* | -- | ⇟ | -- | 0.05 [-0.01; 0.10] | ⨁◯◯◯ |
| Train & educate stakeholders vs no active implementation strategy | 5 | 5† | 75.2%* | -- | ⇟ | -- | 0.03 [0.00; 0.05] | ⨁◯◯◯ |
| Use evaluative & iterative strategies vs no active implementation strategy | 2 | 1 | 40.9% | -- | ⇟ | -- | -0.00 [-0.03; 0.03] | ⨁⨁⨁◯ |
| Provide interactive assistance vs no active implementation strategy | 1 | 1† | -- | -- | ⇟ | -- | 0.06 [-0.05; 0.17] | ⨁⨁◯◯ |
| ***Sensitivity analysis 2, outcome type = Implementation, remove high-risk studies*** | | | | | | | | |
| Implementation strategies vs no active implementation strategy | 7 | 0 | 6.6% | -- | ⇟ | -- | -0.00 [-0.01; 0.01] | ⨁⨁⨁◯ |
| Notes: † Serious risk of bias; * Inconsistency; ⇟ Imprecision; § Publication Bias ; RD: Risk difference; CI: Confidence interval. # The heterogeneity in this comparison can be explained by subgroup analysis or meta-regression.  Sensitivity analysis 1 is the standard meta-analysis for the percentage outcomes of the Patient and Service outcome types; Sensitivity analysis 2 is the standard meta-analysis comparing the presence vs. absence of an implementation strategy | | | | | | | | |

GRADE results for network meta-analysis

| **Name** | **Number of studies** | **Direct evidence** | **Evidence level** | **Indirect evidence** | **Evidence level** | **Network evidence** | **Evidence level** |
| --- | --- | --- | --- | --- | --- | --- | --- |
| Change infrastructure vs Multifaceted implementation strategy | 0 | -- | -- | -0.02 [-0.08; 0.04]*#⇟ | ⨁◯◯◯ very low | -0.02 [-0.08; 0.04]↧ | ⨁◯◯◯ very low |
| Change infrastructure vs Develop stakeholder interrelationships | 0 | -- | -- | -0.06 [-0.21; 0.09]*#⇟ | ⨁◯◯◯ very low | -0.06 [-0.21; 0.09]↧ | ⨁◯◯◯ very low |
| Change infrastructure vs Engage consumers | 0 | -- | -- | 0.01 [-0.06; 0.08]*#⇟ | ⨁◯◯◯ very low | 0.01 [-0.06; 0.08]↧ | ⨁◯◯◯ very low |
| Change infrastructure vs No active implementation strategy | 2 | 0.00 [-0.04; 0.04]†⇟ | ⨁⨁◯◯ low | -- | -- | 0.00 [-0.04; 0.04]↧ | ⨁⨁◯◯ low |
| Change infrastructure vs Support clinicians | 0 | -- | -- | -0.02 [-0.07; 0.02]*#⇟ | ⨁◯◯◯ very low | -0.02 [-0.07; 0.02]↧ | ⨁◯◯◯ very low |
| Change infrastructure vs Train & educate stakeholders | 0 | -- | -- | -0.03 [-0.08; 0.20]*#⇟ | ⨁◯◯◯ very low | -0.03 [-0.08; 0.20]↧ | ⨁◯◯◯ very low |
| Change infrastructure vs Use evaluative & iterative strategies | 0 | -- | -- | 0.01 [-0.05; 0.08]*#⇟ | ⨁◯◯◯ very low | 0.01 [-0.05; 0.08]↧ | ⨁◯◯◯ very low |
| Multifaceted implementation strategy vs Develop stakeholder interrelationships | 0 | -- | -- | -0.04 [-0.19; 0.10]*#⇟ | ⨁◯◯◯ very low | -0.04 [-0.19; 0.10]↧ | ⨁◯◯◯ very low |
| Multifaceted implementation strategy vs Engage consumers | 0 | -- | -- | -0.03 [-0.05; 0.10]*#⇟ | ⨁◯◯◯ very low | -0.03 [-0.05; 0.10]↧ | ⨁◯◯◯ very low |
| Multifaceted implementation strategy vs No active implementation strategy | 3 | 0.02 [-0.03; 0.06]†⇟ | ⨁⨁◯◯ low | -- | -- | 0.02 [-0.03; 0.06]↧ | ⨁⨁◯◯ low |
| Multifaceted implementation strategy vs Support clinicians | 0 | -- | -- | 0.01 [-0.06; 0.04]*#⇟ | ⨁◯◯◯ very low | 0.01 [-0.06; 0.04]↧ | ⨁◯◯◯ very low |
| Multifaceted implementation strategy vs Train & educate stakeholders | 0 | -- | -- | -0.01 [-0.06; 0.04]*#⇟ | ⨁◯◯◯ very low | -0.01 [-0.06; 0.04]↧ | ⨁◯◯◯ very low |
| Multifaceted implementation strategy vs Use evaluative & iterative strategies | 0 | -- | -- | 0.03 [-0.04; 0.10]*#⇟ | ⨁◯◯◯ very low | 0.03 [-0.04; 0.10]↧ | ⨁◯◯◯ very low |
| Develop stakeholder interrelationships vs Engage consumers | 0 | -- | -- | 0.07 [-0.08; 0.23]*#⇟ | ⨁◯◯◯ very low | 0.07 [-0.08; 0.23]↧ | ⨁◯◯◯ very low |
| Develop stakeholder interrelationships vs No active implementation strategy | 1 | 0.06 [-0.08; 0.20]⇟ | ⨁⨁⨁◯ moderate | -- | -- | 0.06 [-0.08; 0.20]↧ | ⨁⨁⨁◯ moderate |
| Develop stakeholder interrelationships vs Support clinicians | 0 | -- | -- | 0.04 [-0.11; 0.18]*#⇟ | ⨁◯◯◯ very low | 0.04 [-0.11; 0.18]↧ | ⨁◯◯◯ very low |
| Develop stakeholder interrelationships vs Train & educate stakeholders | 0 | -- | -- | 0.03 [-0.11; 0.18]*#⇟ | ⨁◯◯◯ very low | 0.03 [-0.11; 0.18]↧ | ⨁◯◯◯ very low |
| Develop stakeholder interrelationships vs Use evaluative & iterative strategies | 0 | -- | -- | 0.07 [-0.08; 0.22]*#⇟ | ⨁◯◯◯ very low | 0.07 [-0.08; 0.22]↧ | ⨁◯◯◯ very low |
| Engage consumers vs No active implementation strategy | 6 | -0.01 [-0.07; 0.05]⇟ | ⨁⨁⨁◯ moderate | -- | -- | -0.01 [-0.07; 0.05]↧ | ⨁⨁⨁◯ moderate |
| Engage consumers vs Support clinicians | 0 | -- | -- | -0.03 [-0.10; 0.03]*#⇟ | ⨁◯◯◯ very low | -0.03 [-0.10; 0.03]↧ | ⨁◯◯◯ very low |
| Engage consumers vs Train & educate stakeholders | 0 | -- | -- | -0.04 [-0.11; 0.03]*#⇟ | ⨁◯◯◯ very low | -0.04 [-0.11; 0.03]↧ | ⨁◯◯◯ very low |
| Engage consumers vs Use evaluative & iterative strategies | 0 | -- | -- | 0.00 [-0.08; 0.08]*#⇟ | ⨁◯◯◯ very low | 0.00 [-0.08; 0.08]↧ | ⨁◯◯◯ very low |
| Support clinicians vs No active implementation strategy | 6 | -0.02 [-0.05; 0.00]†⇟§ | ⨁◯◯◯ very low | -- | -- | -0.02 [-0.05; 0.00]↧ | ⨁◯◯◯ very low |
| Train & educate stakeholders vs No active implementation strategy | 4 | -0.03 [-0.06; -0.00]†§ | ⨁⨁◯◯ low | -- | -- | -0.03 [-0.06; 0.00]↥ | ⨁⨁◯◯ low |
| Use evaluative & iterative strategies vs No active implementation strategy | 1 | 0.01 [-0.04; 0.06]⇟ | ⨁⨁⨁◯ moderate | -- | -- | 0.01 [-0.04; 0.06]↧ | ⨁⨁⨁◯ moderate |
| Support clinicians vs Train & educate stakeholders | 0 | -- | -- | -0.00 [-0.04; 0.03]*#⇟ | ⨁◯◯◯ very low | -0.00 [-0.04; 0.03]↧ | ⨁◯◯◯ very low |
| Support clinicians vs Use evaluative & iterative strategies | 0 | -- | -- | 0.04 [-0.02; 0.09]*#⇟ | ⨁◯◯◯ very low | 0.04 [-0.02; 0.09]↧ | ⨁◯◯◯ very low |
| Train & educate stakeholders vs Use evaluative & iterative strategies | 0 | -- | -- | 0.04 [-0.02; 0.10]*#⇟ | ⨁◯◯◯ very low | 0.04 [-0.02; 0.10]↧ | ⨁◯◯◯ very low |
| Notes: † Serious risk of bias; * Serious Indirectness; # Intransitivity; ⇟ Imprecision; ↥ Direct evidence contributes more; ↧Indirect evidence contributes more; RD: Risk difference; CI: Confidence interval; § Heterogeneity | | | | | | | |

**Supplementary Material 11: Additional results of network meta-analysis**

Network Plot

Each node represents an implementation strategy or the reference condition, “No active implementation strategy”; node size is proportional to the number of study comparisons involving that node. Lines represent direct comparisons between nodes, with line thickness and the number shown on each line indicating the number of studies contributing to that comparison. Notably, this network contains no closed loops; therefore, consistency between direct and indirect evidence could not be assessed.

League Table

|  | Change infrastructure | Multifaceted implementation strategy | Develop stakeholder interrelationships | Engage consumers | No active implementation strategy | Support clinicians | Train & educate stakeholders | Use evaluative & iterative strategies |
| --- | --- | --- | --- | --- | --- | --- | --- | --- |
| Change infrastructure | Change infrastructure | . | . | . |  | . | . | . |
| Multifaceted implementation strategy | -0.0178[-0.0771; 0.0415] | Multifaceted implementation strategy | . | . |  | . | . | . |
| Develop stakeholder interrelationships | -0.0616[-0.2087; 0.0855] | -0.0438[-0.1921; 0.1044] | Develop stakeholder interrelationships | . |  | . | . | . |
| Engage consumers | 0.0109[-0.0617; 0.0834] | -0.0286[-0.0463; 0.1036] | 0.0725[-0.0816; 0.2266] | Engage consumers |  | . | . | . |
| No active implementation strategy | 0.0004[-0.0394; 0.0401] | 0.0182[-0.0258; 0.0622] | 0.0620[-0.0796; 0.2036] | -0.0105[-0.0712; 0.0503] | No active implementation strategy |  |  |  |
| Support clinicians | -0.0238[-0.0707; 0.0231] | 0.0060[-0.0565; 0.0446] | 0.0378[-0.1059; 0.1816] | -0.0346[-0.1003; 0.0310] | -0.0242[-0.0491; 0.0008] | Support clinicians | . | . |
| Train & educate stakeholders | -0.0286[-0.0775; 0.0204] | -0.0108[-0.0632; 0.0417] | 0.0330[-0.1114; 0.1775] | -0.0394[-0.1066; 0.0277] | -0.0290[-0.0576; -0.0004] | -0.0048[-0.0427; 0.0331] | Train & educate stakeholders | . |
| Use evaluative & iterative strategies | 0.0120[-0.0513; 0.0753] | 0.0298[-0.0362; 0.0959] | 0.0737[-0.0763; 0.2236] | 0.0012[-0.0770; 0.0794] | 0.0116[-0.0376; 0.0609] | 0.0358[-0.0194; 0.0910] | 0.0406[-0.0164; 0.0976] | Use evaluative & iterative strategies |

Heterogeneity assessment results

**Q statistics to assess homogeneity / consistency**

|  | Q | df | p-value |
| --- | --- | --- | --- |
| Total | 49.64 | 16 | < 0.0001 |
| Within designs | 49.64 | 16 | < 0.0001 |
| Between designs | 0 | 0 | -- |

**Design-specific decomposition of within-designs Q statistic**

| Design | Q | df | p-value |
| --- | --- | --- | --- |
| No active implementation strategy:Engage consumers | 7.1 | 5 | 0.21 |
| No active implementation strategy:Support clinicians | 25.93 | 5 | < 0.0001 |
| No active implementation strategy:Train & educate stakeholders | 14.51 | 3 | 0.0023 |
| No active implementation strategy:Change infrastructure | 1.25 | 1 | 0.263 |
| No active implementation strategy:Multifaceted implementation strategy | 0.74 | 2 | 0.657 |

**Q statistic to assess consistency under the assumption of a full design-by-treatment interaction random effects model**

|  | Q | df | p-value | tau.within | tau2.within |
| --- | --- | --- | --- | --- | --- |
| Between designs | 0 | 0 | -- | 0.0223 | 0.0005 |

**Supplementary Material 12: Secondary outcome (service and patient outcomes) results**

Service outcome

Patient outcome

**Supplementary Material 13: Effects of implementation strategies in included studies not eligible for meta-analysis**

Dichotomous outcomes

| **Reference** | **Reason for exclusion of meta-analysis** | **Outcome** |
| --- | --- | --- |
| Diaz et al. 2021 US | Missing data on total number of participants in groups | Implementation outcome (Uptake) (+): PCPs who elected to have performance-based financial incentives referred more patients for teleophthalmology than those who did not make this election. |
| Ziemer et al. 2006 US | Missing data on number of events in each group | Implementation outcome (Fidelity) (+): Healthcare provider behavior improved significantly more for the feedback and reminder intervention groups for each 6-month time period than for reminder alone group (p<0.01). |
| Leung et al. 2017 Australia | Missing data on total number of participants in groups | Implementation outcome (Fidelity) (-): Neither intervention had a significant impact on duplication alert rate (Feedback: 80.8% vs. 77.8% of orders, Training: 77.5% vs. 76.5% of orders; all p>0.05). |

Continuous outcomes

Continuous outcomes are presented descriptively in their original units, as reported in the included studies; no standardized effect sizes were calculated and no quantitative synthesis was undertaken. They were considered for possible synthesis but were not pooled with the percentage-based risk differences. The available outcomes were reported across distinct outcome domains, including implementation uptake and fidelity, patient-centeredness, patient satisfaction and symptomatology, and service efficiency. Within and across these domains, studies used non-comparable measures and analytic summaries, including individual mean changes, post-intervention scores, annualized eGFR slopes, and numbers of hospitalizations, often at different follow-up times or across multiple intervention arms. Although HbA1c was reported in more than one study, the available data were reported in different forms and did not provide a consistent, adequately reported contrast for a common standardized analysis. We therefore considered that a pooled standardized mean difference would not provide a clinically interpretable common estimate or be directly comparable with the percentage-based risk-difference analyses. These outcomes were therefore summarized narratively.

| **Reference** | **Outcome** |
| --- | --- |
| 117 Phillips et al. 2005 US | Patient outcomes (Symptomatology): Participants’ changes in HbA1C in the feedback + reminder group was -0.56 (p<0.0001) and -0.3 (p=0.0134) in the reminders only group; Participants’ changes in sBP in the feedback + reminder group was -3.4 (p=0.0037) and 1.2 (p=0.3477) in the reminders only group; Participants’ changes in LDL cholesterol in the feedback + reminder group was -18.2 (p<0.0001) and -15.23 (p<0.0001) in the reminders only group. |
| 118 Carroll et al. 2018 US | Patient outcomes (Symptomatology): Difference in eGFR slope was 0.93 (p<0.01) for intervention group and was -0.94 (p<0.01) for control group. |
| 101 Gordon et al. 2022 US | 1. Service outcomes (Patient centeredness) (+): Patients rated higher towards providers’ empathy (p=0.04) and provider-patient communication (p=0.05).  2. Patient outcomes (Symptomatology) (-): Post-visit HbA1c was not statistically different. |
| 109 Peels et al. 2024 Netherland | Implementation outcomes (Uptake) (-): Intention to implement Active Plus within the next year is 3.4 at baseline, 3.57 at interim, and 2.3 at the final measurement (p=0.023). |
| 124 Subramanian et al. 2004 US | Patient outcomes (Satisfaction) (+): Intervention patients were more satisfied with their physicians (p=0.02) and primary care visit (p=0.02).  Service outcomes (Efficiency) (-): No significantly differences in the number of outpatient visits between intervention and control patients (6.7 vs. 7.1 visits, p=0.48). Intervention patients had more all-cause hospitalizations at 6 months (1.5 vs. 0.7 hospitalizations, p=0.0002) and 12 months (2.3 vs. 1.7 hospitalizations, p=0.05). |
| 131 Schroter et al. 2011 UK | Implementation outcome (Fidelity) (-): Doctors’ test scores increased from 47.4% to 59.0% for intervention group and 47.3% to 60.1% for control group (p=0.186). |
| 105 Sarna et al. 2012 US | Implementation outcomes (Fidelity) (+/-): Mean improvement scores were not significantly higher in Ask (p=0.61), Advise (p=0.99), Assess (p=0.36), Assist (p=0.58), Arrange (0.30), but only in Referral (p=0.04) to a Quitline for the Help Smokers Quit group. |
| 132 Learmonth et al. 2025 Australia | Implementation outcomes (Fidelity) (+/-):Exercise prescription rates varied, increasing for those with fewer clients with MS, but decreasing for those with more clients with MS. |

Of the eight studies reporting continuous outcomes, three also contributed percentage-based implementation outcomes to the primary meta-analysis (Subramanian et al., and Schroter et al., Sarna et al.). Of the remaining five studies, three reported patient or service outcomes only, whereas the two reporting implementation outcomes did not provide poolable comparative data and/or used time-series designs.

**Supplementary Material 14: Meta-regression**

Seventeen study-level variables were initially examined in separate univariable random-effects meta-regression models to explore their individual associations with heterogeneity. To formalize subsequent model building, we performed bidirectional stepwise selection using the Akaike information criterion corrected for small sample sizes (AICc). Lower AICc values indicate a better balance between model fit and complexity.

All candidate models were fitted using the same 23 study comparisons. The procedure began with an intercept-only model. At each forward step, each remaining moderator was added separately, and a moderator was retained only when it reduced the AICc by at least two points. Backward deletion was considered using the same criterion. To reduce overfitting, models were restricted to a maximum of two moderator terms and were required to retain at least 10 residual degrees of freedom. Maximum-likelihood estimation was used for model selection, and the selected model was refitted using restricted maximum likelihood estimation for reporting.

The complete candidate-model trace, including AICc, Delta AICc, and the selection decision at each step, is presented in following table.

| **Selection stage** | **Current retained model** | **Candidate model** | **AICc** | **Delta AICc** | **Decision** |
| --- | --- | --- | --- | --- | --- |
| Start |  | Null model (intercept-only) | -62.91 |  | Reference model |
| Round 1 (forward) | Null model (intercept-only) | Outcome_subtype | -60.81 | 2.10 | Not selected |
| Round 1 (forward) | Null model (intercept-only) | Role | -59.53 | 3.39 | Not selected |
| Round 1 (forward) | Null model (intercept-only) | Age | -60.39 | 2.53 | Not selected |
| Round 1 (forward) | Null model (intercept-only) | Sex | -59.47 | 3.45 | Not selected |
| Round 1 (forward) | Null model (intercept-only) | Country | -60.34 | 2.58 | Not selected |
| Round 1 (forward) | Null model (intercept-only) | Urban/rural | -55.75 | 7.17 | Not selected |
| Round 1 (forward) | Null model (intercept-only) | Places | -57.39 | 5.53 | Not selected |
| Round 1 (forward) | Null model (intercept-only) | Public/private | -63.65 | -0.74 | Not selected |
| Round 1 (forward) | Null model (intercept-only) | HCP roles | -59.06 | 3.85 | Not selected |
| Round 1 (forward) | Null model (intercept-only) | Disease category | -75.31 | -12.40 | **Selected** |
| Round 1 (forward) | Null model (intercept-only) | Data collection method | -58.48 | 4.43 | Not selected |
| Round 1 (forward) | Null model (intercept-only) | DHTs category | -61.95 | 0.96 | Not selected |
| Round 1 (forward) | Null model (intercept-only) | new HCP responsibilities? | -60.72 | 2.19 | Not selected |
| Round 1 (forward) | Null model (intercept-only) | new HCP role? | -59.32 | 3.59 | Not selected |
| Round 1 (forward) | Null model (intercept-only) | whether HCP co-create | -60.64 | 2.28 | Not selected |
| Round 1 (forward) | Null model (intercept-only) | No_of_components | -62.77 | 0.15 | Not selected |
| Round 1 (forward) | Null model (intercept-only) | Dosage | -63.67 | -0.75 | Not selected |
| Round 2 (forward) | Disease category | Disease category + Outcome_subtype | -69.51 | 5.80 | Not selected |
| Round 2 (forward) | Disease category | Disease category + Role | -69.46 | 5.86 | Not selected |
| Round 2 (forward) | Disease category | Disease category + Age | -70.81 | 4.51 | Not selected |
| Round 2 (forward) | Disease category | Disease category + Sex | -67.75 | 7.56 | Not selected |
| Round 2 (forward) | Disease category | Disease category + Country | -71.63 | 3.68 | Not selected |
| Round 2 (forward) | Disease category | Disease category + Urban/rural | -63.22 | 12.09 | Not selected |
| Round 2 (forward) | Disease category | Disease category + Places | -64.97 | 10.34 | Not selected |
| Round 2 (forward) | Disease category | Disease category + Public/private | -64.83 | 10.49 | Not selected |
| Round 2 (forward) | Disease category | Disease category + HCP roles | -71.44 | 3.88 | Not selected |
| Round 2 (forward) | Disease category | Disease category + Data collection method | -68.78 | 6.53 | Not selected |
| Round 2 (forward) | Disease category | Disease category + DHTs category | -63.06 | 12.25 | Not selected |
| Round 2 (forward) | Disease category | Disease category + new HCP responsibilities? | -71.75 | 3.56 | Not selected |
| Round 2 (forward) | Disease category | Disease category + new HCP role? | -69.97 | 5.34 | Not selected |
| Round 2 (forward) | Disease category | Disease category + whether HCP co-create | -71.26 | 4.05 | Not selected |
| Round 2 (forward) | Disease category | Disease category + No_of_components | -71.78 | 3.53 | **Best in round; not selected** |
| Round 2 (forward) | Disease category | Disease category + Dosage | -68.24 | 7.07 | Not selected |

In the bidirectional stepwise selection based on AICc, disease category was selected as the most parsimonious moderator model. No additional moderator improved model fit after disease category was included. The results for the final model can be found below:

Model results part one:

Mixed-Effects Model (k = 23; tau^2 estimator: REML)

| logLik | deviance | AIC | BIC | AICc |
| --- | --- | --- | --- | --- |
| 30.0224 | -60.0448 | -50.0448 | -45.3227 | -45.4295 |

Test for Residual Heterogeneity:

QE(df = 19) = 32.4460, p-val = 0.0278

Test of Moderators (coefficients 2:4):

QM(df = 3) = 84.8930, p-val < .0001

Model results part two:

|  | estimate | se | zval | pval | ci.lb | ci.ub |
| --- | --- | --- | --- | --- | --- | --- |
| intrcpt | 0.0492 | 0.0048 | 10.2785 | <.0001 | 0.0398 | 0.0586 |
| `Disease category`DM | -0.0446 | 0.0074 | -6.0218 | <.0001 | -0.0591 | -0.0301 |
| `Disease category`Mixed | -0.0329 | 0.0093 | -3.5271 | 0.0004 | -0.0512 | -0.0146 |
| `Disease category`Others | -0.0482 | 0.0053 | -9.1349 | <.0001 | -0.0585 | -0.0378 |

**Supplementary Material 15: Subgroup analysis**

Healthcare provider (HCP) behaviours (Test for subgroup differences: p = < 0.0001)

Disease category (Test for subgroup differences: p = 0.0667)

Number of components in a strategy (Test for subgroup differences: p = 0.0170)

Frequency of strategy delivery (Test for subgroup differences: p = 0.1771)

**Supplementary Material 16: Publication bias**

Funnel plot for the standard meta-analysis in the primary analysis

Results of the Egger test:

Linear regression test of funnel plot asymmetry

Test result: t = 1.01, df = 21, p-value = 0.3249

Bias estimate: 0.5945 (SE = 0.5898)

Details:

- multiplicative residual heterogeneity variance (tau^2 = 5.3493)

- predictor: standard error

- weight: inverse variance

- reference: Egger et al. (1997), BMJ

Funnel plot for the network meta-analysis

Note:

[1] "No active implementation strategy"

[2] "Train & educate stakeholders"

[3] "Multifaceted implementation strategy"

[4] "Support clinicians"

[5] "Change infrastructure"

[6] "Develop stakeholder interrelationships"

[7] "Engage consumers"

[8] "Use evaluative & iterative strategies"

Egger test results: P = 0.10

**Supplementary Material 17: Sensitivity Analysis**

Sensitivity analysis 1: Reclassification of multifaceted implementation strategies by constituent ERIC categories

Sensitivity analysis 2: (1) Exclusion of studies with a high risk of bias

Sensitivity analysis 2: (2) Including studies with low risk of bias only

Sensitivity analysis 3: Leave-one-out analysis

|  |  | RD[95%CI] | p-value | tau^2 | tau | I^2 |
| --- | --- | --- | --- | --- | --- | --- |
| Omitting 102 | Bailey 2021 | 0.0171 [ 0.0011; 0.0331] | 0.036 | 0.0006 | 0.0238 | 82.00% |
| Omitting 103 | Filardo 2009 | 0.0180 [ 0.0033; 0.0328] | 0.0166 | 0.0005 | 0.022 | 82.10% |
| Omitting 104 | Gil-Guillén 2015 | 0.0068 [-0.0008; 0.0144] | 0.0808 | 0 | 0.0066 | 52.90% |
| Omitting 105 | Sarna 2012 | 0.0157 [ 0.0018; 0.0297] | 0.0272 | 0.0004 | 0.0208 | 81.90% |
| Omitting 106 | Estrada 2011 | 0.0167 [ 0.0016; 0.0319] | 0.0305 | 0.0005 | 0.0226 | 82.10% |
| Omitting 107 | Leonard 2024 | 0.0160 [ 0.0019; 0.0302] | 0.0263 | 0.0004 | 0.0211 | 82.10% |
| Omitting 111 | van Wyk 2007 | 0.0134 [ 0.0007; 0.0261] | 0.0383 | 0.0003 | 0.018 | 78.90% |
| Omitting 112 | Rosenbloom 2005 | 0.0189 [ 0.0032; 0.0346] | 0.018 | 0.0005 | 0.0228 | 75.10% |
| Omitting 113 | Strom 2010 | 0.0181 [ 0.0038; 0.0324] | 0.0131 | 0.0005 | 0.0212 | 82.00% |
| Omitting 114 | McAlister 2009 | 0.0161 [ 0.0020; 0.0302] | 0.0248 | 0.0004 | 0.0211 | 82.10% |
| Omitting 115 | Derose 2005 | 0.0180 [ 0.0019; 0.0341] | 0.0285 | 0.0006 | 0.024 | 82.20% |
| Omitting 119 | Manns 2012 | 0.0189 [ 0.0035; 0.0343] | 0.0164 | 0.0005 | 0.0224 | 81.80% |
| Omitting 120 | Roumle 2006 | 0.0170 [ 0.0027; 0.0312] | 0.0196 | 0.0005 | 0.0214 | 82.20% |
| Omitting 121 | Loo 2011 | 0.0160 [ 0.0016; 0.0304] | 0.0298 | 0.0005 | 0.0215 | 82.10% |
| Omitting 122 | Feldstein 2006 | 0.0173 [ 0.0032; 0.0314] | 0.0164 | 0.0004 | 0.0212 | 82.00% |
| Omitting 123 | Simon 2006 | 0.0175 [ 0.0027; 0.0322] | 0.0201 | 0.0005 | 0.022 | 82.20% |
| Omitting 124 | Subramanian 2004 | 0.0167 [ 0.0016; 0.0318] | 0.0307 | 0.0005 | 0.0226 | 82.10% |
| Omitting 125 | Heiman 2004 | 0.0155 [ 0.0016; 0.0295] | 0.0293 | 0.0004 | 0.0207 | 81.90% |
| Omitting 126 | Blecker 2019 | 0.0146 [ 0.0009; 0.0283] | 0.0371 | 0.0004 | 0.02 | 81.60% |
| Omitting 128 | Persell 2008 | 0.0163 [ 0.0022; 0.0303] | 0.0234 | 0.0004 | 0.0211 | 82.10% |
| Omitting 129 | Kenealy 2005 | 0.0178 [ 0.0037; 0.0319] | 0.0135 | 0.0004 | 0.0211 | 81.80% |
| Omitting 130 | Stamenova 2023 | 0.0173 [ 0.0032; 0.0314] | 0.016 | 0.0004 | 0.0211 | 81.90% |
| Omitting 131 | Schroter 2011 | 0.0192 [ 0.0048; 0.0336] | 0.0089 | 0.0004 | 0.0205 | 81.60% |
| Pooled estimate |  | 0.0166 [ 0.0026; 0.0306] | 0.0202 | 0.0004 | 0.0210 | 81.3% |

Reference:

1.	Borges do Nascimento IJ, Abdulazeem HM, Vasanthan LT, Martinez EZ, Zucoloto ML, Østengaard L, et al. The global effect of digital health technologies on health workers' competencies and health workplace: an umbrella review of systematic reviews and lexical-based and sentence-based meta-analysis. Lancet Digit Health. 2023;5(8):e534-e44.

2.	Waltz TJ, Powell BJ, Matthieu MM, Damschroder LJ, Chinman MJ, Smith JL, et al. Use of concept mapping to characterize relationships among implementation strategies and assess their feasibility and importance: results from the Expert Recommendations for Implementing Change (ERIC) study. Implementation Science. 2015;10(1):109.

3.	Proctor E, Silmere H, Raghavan R, Hovmand P, Aarons G, Bunger A, et al. Outcomes for implementation research: conceptual distinctions, measurement challenges, and research agenda. Adm Policy Ment Health. 2011;38(2):65-76.
